# Syntheses, Structures, and Magnetic Properties of a Series of Heterotri-, Tetra- and Pentanuclear Ln^III^–Co^II^ Compounds

**DOI:** 10.3390/polym11020196

**Published:** 2019-01-23

**Authors:** Yun Xu, Feng Luo, Ji-Min Zheng

**Affiliations:** 1 Department of Chemistry, Nankai University, Tianjin 300071, China; xuyun88@163.com (Y.X.); ecitluofeng@163.com (F.L.); 2 College of Chemistry and Materials Science, HuaiBei Normal University, Huaibei 235000, China

**Keywords:** heterometallic, pentanuclear, magnetic properties

## Abstract

Three types of Ln(III)–Co(II) heterometallic compounds, LnCo_2_(L1)_7_(bipy)_2_ (Ln = Pr-**1**, Eu-**2**, Sm-**3**, Gd-**4**, Tb-**5**, Dy-**6**) (L1 = 4-chlorobenzoate, bipy = 2,2′-bipyridine), Ln_2_Co_2_(L_2_)_10_(bipy)_2_ (Ln = Sm-**7**, Gd-**8**, Tb-**9**, Dy-**10**, Ho-**11**, Er-**12**, Yb-**13**), (L2 = 2,4-dichlorobenzoate, bipy = 2,2′-bipyridine, phen = 1,10-phenanthroline), and Ln_2_Co_3_(L1)_12_(bipy)_2_ (Ln = Ho-**14**, Er-**15**, Yb-**16**), were synthesized under hydrothermal conditions and characterized by single crystal X-ray diffraction, IR spectroscopy and magnetic measurements. Structural analyses revealed that **14**–**16** take on a unique linear pentanuclear structural motif. Interestingly, the Ho-containing compound **14** exhibits magnetic relaxation behavior.

## 1. Introduction

Heterometallic compounds containing both d-block transition and f-block lanthanide metal ions have been intensively investigated due to their impressive structural diversity and their exploitable applications in magnetism, luminescent materials and molecular adsorption [1,2,3,4,5,6]. Because of the the different nature between lanthanide and transition metal ions, the synthesis of a compound containing both of them is difficult compared with the synthesis of a compound containing only one of them. With respect to lanthanide ions, they have a high affinity for binding to hard donors like the O atom. While most transition metal ions prefer to coordinate to soft donors like the N atom, many strategies are used for synthesizing compounds containing both lanthanide and transition metal ions, for example, selecting proper multidentate ligands containing both N- and O-donor atoms, such as pyridine-2,5-dicarboxylic acid, pyridine-2,6-dicarboxylic acid, nicotinic acid, isonicotinic acid and iminodiacetate ligand [7,8,9,10]. The use of polycarboxylate and bipyridyl ligands has also proved to be a constructive way to synthesis the **3**d–**4**f heterometallic compounds [11]. The magnetic property of the **3**d–**4**f heterometallic compound is also a challenge—because the orbital contributions of most f electron pairs and the influence of the crystal field effects have to be considered, the analyses of magnetic interactions among Ln(III) and transition metal ions become very difficult [12,13,14]. As the ground state of the Gd(III)ion is ^8^S_7/2_, the spin-orbit coupling effect is absent, and the influence of the ligand field can be neglected. As a consequence, most studies on **3**d–**4**f structures have involved Gd(III)–M(II) or Gd(III)–M(III) systems [15,16,17,18,19], with examples of the Ln(III)–Co(II) system being much less documented [20].

Inspired by the above mentioned information, we successfully synthesized three series of trinuclear, tetranuclear and pentanuclear Ln(III)–Co(II) ions under hydrothermal conditions by selecting 4-chlorobenzoic acid/2,4-dichlorobenzoic acid (L1/L2), bipy as ligands.

## 2. Experimental Section

### 2.1. Materials and Physical Measurements

All reagents and solvents used for synthesis and analysis are commercially available and were used as received. IR spectra were taken on a Perkin–Elmer spectrum. One FT-IR spectrometer (Thermo Fisher Scientific, Waltham, MA, USA) was used in the 4000–400 cm^−1^ region with KBr pellets. Elemental analyses for C, H and N were carried out on a Model 2400II, Perkin–Elmer elemental analyzer (Perkin Elmer, Akron, OH, USA). The magnetic susceptibility measurements of the polycrystalline samples were measured over the temperature range of 2–300 K with a Quantum Design MPMSXL7 SQUID magnetometer (Quantum Design, San Diego, CA, USA) using an applied magnetic field of 1000 Oe. The ac magnetic data were measured on a PPMS-9 ACMS magnetometer (Quantum Design, San Diego, CA, USA).

### 2.2. Preparation

All starting materials, except for LnCl_3_·6H_2_O, are commercially available and were used without further purification. LnCl_3_·6H_2_O was prepared from the reaction of lanthanide oxide with a concentrated hydrochloric acid solution.

#### 2.2.1. Preparation of PrCo_2_(L1)_7_(bipy)_2_ (**1**)

A mixture containing PrCl_3_·6H_2_O (0.07 g, 0.2 mmol) Co(NO_3_)_2_·6H_2_O (0.06 g, 0.2 mmol), 4-chlorobenzoate (L1; 0.16 g, 1.0 mmol), bipy (0.03 g, 0.2 mmol), Na_2_CO_3_ (0.05 mg, 0.5 mmol), water (10 mL) and ethanol (5 mL) was sealed in a Teflon-lined stainless steel vessel (23 mL), which was heated at 160 °C for 3 days and then cooled to room temperature at a rate of 5 °C/h. Red block crystals of **1** were obtained and picked out, washed with distilled water and dried in air. Yield: 32% (based on Co(II)). Elemental analysis for C_69_H_44_Cl_7_Co_2_N_4_O_14_Pr (%) Calcd.: C 49.92, H 2.67, N 3.38. Found: C 49.96, H 2.66, N 3.39. IR (KBr, cm^−1^): 1611(vs), 1563(m), 1409(vs), 1166(m), 1094(m), 1010(m), 846(m), 768(s).

#### 2.2.2. Preparation of LnCo_2_(L1)_7_(bipy)_2_ (Ln = Eu, Sm, Gd, Tb, Dy) (**2**–**6**)

The same synthetic procedure as that of **1** was used except that PrCl_3_·6H_2_O was replaced by LnCl_3_·6H_2_O (0.06–0.08 g, 0.2 mmol) (Ln = Eu, Sm, Gd, Tb, Dy). Red block crystals of **2**–**6** were obtained and picked out, washed with distilled water and dried in air. Yield: 41% of **2**. (based on Co(II)). Elemental analysis for C_69_H_44_Cl_7_Co_2_N_4_O_14_Eu (%): Calcd.: C 49.59, H 2.65, N 3.35; Found: C 49.54, H 2.67, N 3.32. IR (KBr, cm^−1^): 1610(vs), 1560(m), 1409(vs), 1165(m), 1093(m), 1011(m), 847(m), 768(s). Yield: 46% of **3** (based on Co(II)). Elemental analysis for C_69_H_44_Cl_7_Co_2_N_4_O_14_Sm(%) Calcd.: C 49.64, H 2.66, N 3.36. Found: C 49.67, H 2.65, N 3.38. IR (KBr, cm^−1^): 1611(vs), 1561(m), 1409(vs), 1165(m), 1094(m), 1010(m), 846(m), 768(s). Yield: 52% of **4** (based on Co(II)). Elemental analysis for C_69_H_44_Cl_7_Co_2_N_4_O_14_Gd (%): Calcd.: C, 49.44; H, 2.65; N, 3.34. Found: C, 49.48; H, 2.69; N, 3.36. IR (KBr, cm^−1^): 1610(vs), 1560(m), 1408(vs), 1165(m), 1093(m), 1011(m), 847(m), 768(s). Yield: 47% of **5** (based on Co(II)). Elemental analysis for C_69_H_44_Cl_7_Co_2_TbN_4_O_14_ (%) Calcd.: C, 49.39; H, 2.64; N, 3.34. Found: C, 49.44; H, 2.61; N, 3.37. IR (KBr, cm^−1^): 1611(vs), 1561(m), 1408(vs), 1165(m), 1093(m), 1011(m), 846(m), 768(s). Yield: 47% of **6** (based on Co(II)). Elemental analysis for C_69_H_44_Cl_7_Co_2_N_4_O_14_Dy (%) Calcd.: C49.28, H 2.64, N 3.33. Found: C 49.29, H 2.64, N 3.31. IR (KBr, cm^−1^): 1611(vs), 1561(m), 1407(vs), 1165(m), 1095(m), 1010(m), 846(m), 770(s).

#### 2.2.3. Preparation of Ln_2_Co_2_(L2)_10_(bipy)_2_ (Ln = Sm, Gd, Tb, Dy, Ho, Er, Yb) (**7**–**13**)

The same synthetic procedure as that for **1** was used except that PrCl_3_·6H_2_O was replaced by LnCl_3_·6H_2_O (0.07–0.09 g, 0.2 mmol), L1 was replaced by 2,4-dichlorobenzoate (L2; 0.19 g, 1.0 mmol). Red block crystals of **7**–**13** were obtained and picked out, washed with distilled water and dried in air. Yield: 37% of **7** (based on Co(II)). Elemental analysis for **7**, C_90_H_46_Cl_20_Co_2_N_4_O_20_Sm_2_ (%) Calcd.: C, 41.09; H, 1.76; N, 2.13. Found: C, 41.03; H, 1.74; N, 2.11. IR (KBr, cm^−1^): 3090(m), 1593(vs), 1476(m), 1418(s), 1105(m), 1053(m), 863(m), 790(m). Yield: 58% of **8** (based on Co(II)). Elemental analysis for **8**, C_90_H_46_Cl_20_Co_2_N_4_O_20_Gd_2_ (%) Calcd.: C, 40.87; H, 1.75; N, 2.12. Found: C, 40.83; H, 1.71; N, 2.17. IR (KBr, cm^−1^): 3090(m), 1593(vs), 1476(m), 1418(s), 1097(m), 1046(m), 863(m), 790(s). Yield: 46% of **9** (based on Co(II)). Elemental analysis for **9**, C_90_H_46_Cl_20_Co_2_N_4_O_20_Tb_2_ (%) Calcd.: C, 40.82; H, 1.75; N, 2.12. Found: C, 40.79; H, 1.71; N, 2.14. IR (KBr, cm^−1^): 3090(m), 1593(vs), 1476(m), 1418(s), 1097(m), 1046(m), 863(m), 790(s). Yield: 45% of **10** (based on Co(II)). Elemental analysis for **10** C_90_H_46_Cl_20_Co_2_N_4_O_20_Dy_2_ (%) Calcd.: C, 40.71; H, 1.75; N, 2.11. Found: C, 40.67; H, 1.71; N, 2.14. IR (KBr, cm^−1^): 3090(m), 1593(vs), 1476(m), 1418(s), 1097(m), 1046(m), 863(m), 789(s). Yield: 48% of **11**(based on Co(II)). Elemental analysis for **11**, C_90_H_46_Cl_20_Co_2_N_4_O_20_Ho_2_ (%) Calcd.: C, 40.64; H, 1.74; N, 2.11. Found: C, 40.61; H, 1.69; N, 2.15. IR (KBr, cm^−1^): 3090(m), 1593(vs), 1475(m), 1418(s), 1104(m), 1053(m), 863(m), 784(m). Yield: 51% of **12** (based on Co(II)). Elemental analysis for **12**, C_90_H_46_Cl_20_Co_2_N_4_O_20_Er_2_ (%) Calcd.: C, 40.56; H, 1.74; N, 2.10. Found: C, 40.52; H, 1.71; N, 2.13. IR (KBr, cm^−1^): 3090(m), 1593(vs), 1476(m), 1418(s), 1097(m), 1046(m), 863(m), 789(s). Yield: 36% of **13** (based on Co(II)). Elemental analysis for **13**, C_90_H_46_Cl_20_Co_2_Yb_2_N_4_O_20_ (%)Calcd.: C 40.39, H 1.73, N2.09. Found: C 40.35, H 1.77, N 2.13. IR (KBr, cm^−1^): 3090(m), 1593(vs), 1476(m), 1417(s), 1097(m), 1046(m), 865(m), 790(s).

#### 2.2.4. Preparation of Ln_2_Co_3_(L1)_12_(bipy)_2_ (Ln = Ho, Er, Yb) (**14**–**16**)

The same synthetic procedure as that for **1** was used except that PrCl_3_·6H_2_O was replaced by LnCl_3_·6H_2_O (Ln = Ho, Er, Yb; 0.09 g, 0.2 mmol). Red block crystals of **14**–**16** were obtained and picked out, washed with distilled water and dried in air. Yield: 51% of **14** (based on Co(II)). Elemental analysis for **14**, C_104_H_64_Cl_12_Co_3_Ho_2_N_4_O_24_ (%) Calcd.: C46.51, H 2.40, N 2.09; Found: C 46.53, H 2.39, N 2.11. IR (KBr, cm^−1^): 1593(vs), 1540(m), 1407(vs), 1281(m), 1171(m), 1093(m), 1011(m), 858(m), 774(m). Yield: 55% of **15** (based on Co(II)). Elemental analysis for **15**, C_52_H_32_Cl_6_Co_1.5_ErN_2_O_12_ (%) Calcd.: C 46.43, H 2.40, N 2.08. Found: C 46.41, H 2.43, N 2.12. IR (KBr, cm^−1^): 1593(vs), 1540(m), 1411(vs), 1280(m), 1171(m), 1094(m), 1010(m), 858(m), 773(m). Yield: 38% of **16** (based on Co(II)). Elemental analysis for **16**, C_104_H_64_Cl_12_Co_3_Yb_2_N_4_O_24_ (%) Calcd.: C 46.23, H 2.39, N 2.07; Found: C 46.25, H 2.32, N 2.04. IR (KBr, cm^−1^): 1593(vs), 1540(m), 1413(s), 1276(m), 1173(m), 1089(m), 1010(m), 857(m), 768(s). 

### 2.3. X-ray Crystallographic Determinations

Single crystal analyses were performed on the RAXIS-RAPID AUTO CCD diffractometer systems (MoKα radiation, λ = 0.71073 Å) for **1**–**18**. All data were corrected for absorption by the semiempirical method using the SADABS program. The program SAIN was applied for integration of the diffraction profiles [21]. Data analyses were carried out with the program XPREP. The structures were solved with the direct method using SHELXS-2014 followed by structure refinement on F^2^ with the program SHELXL-2014 [22]. All nonhydrogen atoms were refined anisotropically. Aromatic hydrogen atoms were assigned to calculated positions with isotropic thermal parameters. Crystal data and experimental details are summarized in Table 1, Table 2, Table 3, Table 4 and Table 5. Selected bonds and angles are listed in Tables S1–S3.

## 3. Result and Discussion

### 3.1. Description of the Structures

#### 3.1.1. Crystal Structures of Compounds **1**–**6**

**Figure 1:**
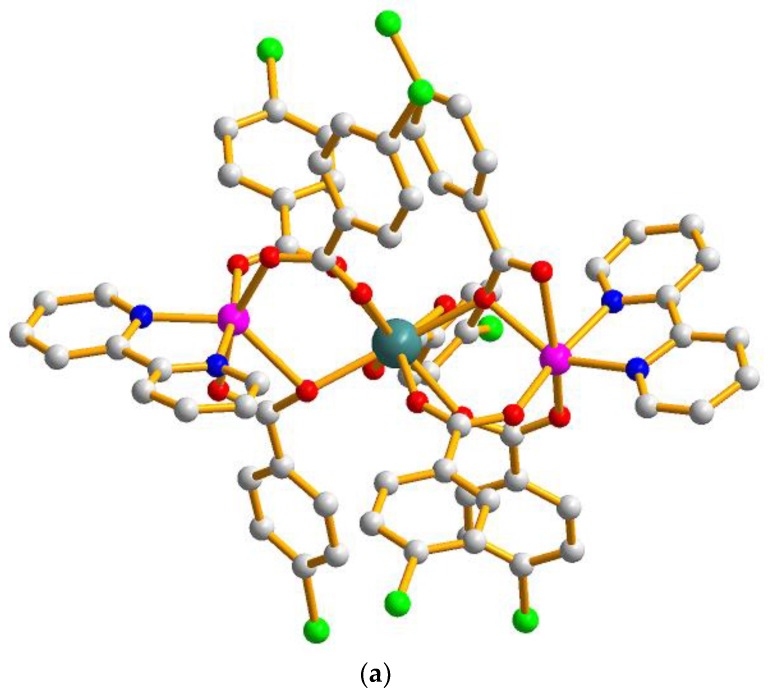

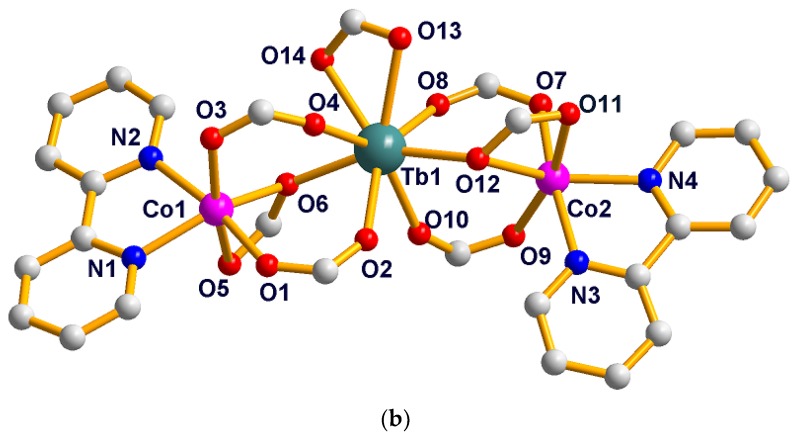
(**a**) The molecule structure of the trinuclear [TbCo_2_(L1)_7_(bipy)_2_] of **5**. Hydrogen atoms are omitted for clarity. (**b**) The coordination environments of the metal centers in **5**.

**Figure 2:**
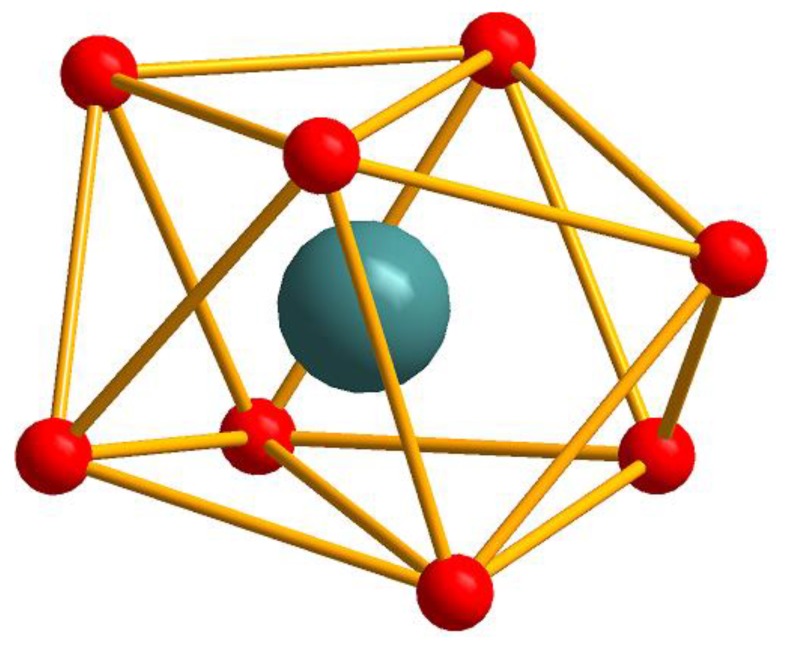
The distorted dodecahedron coordination geometry of Tb(III) ion in **5** with the TbO_8_ sphere.

**Scheme 1:**
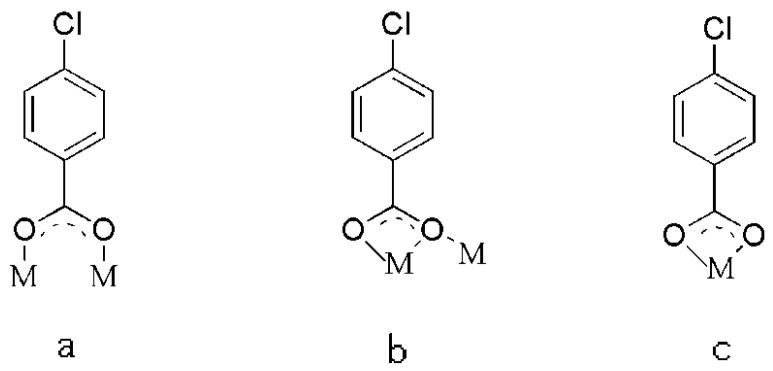
The coordinated modes of the L1 ligands in **5**.

The single crystal X-ray analysis revealed that compounds **1**–**6** are isostructural. They crystallize in the triclinic *P*$\overset{-}{1}$ space group and consist of quasi linear trinuclear metal ions. Hence, only the structure of **5** is described in detail. As described in Figure 1a, the structure of **5** contains one Tb(III) ion (Tb1), two crystallographically independent Co(II) ions (Co1 and Co2), and seven 4-chlorobenzoate ligands and two crystallographically independent bipy ligands. The coordination environment of the metal ions (Tb1, Co1 and Co2) in **5** is presented in Figure 1b. Co1 is six-coordinated with distorted octahedron geometry, composed of four carboxylic O atoms (O1, O3, O5, O6) from three different L1 ligands and two N atoms (N1, N2) from one bipy ligand (Co1–O = 2.026(2)–2.370(3) Å, Co1–N = 2.094(3)–2.154(3) Å). The Co2 ion is also six-coordinated with a slightly distorted octahedral coordination environment, composed of four carboxylic O atoms (O7, O9, O11, O12) from three different L1 ligands and two N atoms (N3, N4) from one bipy ligand (Co2–O = 1.980(3)–2.401(3) Å, Co2–N = 2.098(3)–2.128(3) Å). The Tb1 ion is located on the crystallographic inversion centre and is eight-coordinated with the distorted dodecahedron coordination geometry, as depicted in Figure 2, composed of eight carboxylic O atoms from seven different L1 ligands (Tb1-O = 2.326(2)–2.497(2) Å). Two bridging *μ-1,2* [bis(monodentate)] carboxylate groups and one *μ-1,1,2* [bidentate-monodentate] carboxylate group link the Tb(III) ion and Co(II) together (Tb1–Co = 3.7977(13)–3.8508(9) Å, Co1–Tb1–Co2 = 150.573(13)°). There are three coordinated modes of the L1 ligands in **5**: two bridging (a) *μ-1,2* [bis(monodentate)] and (b) *μ-1,1,2* [bidentate-monodentate] and chelating (c) bidentate, as depicted in Scheme 1. Two bipy ligands are located on the two ends as the terminal ligands.

#### 3.1.2. Crystal Structures of Compounds 7–13

**Figure 3:**
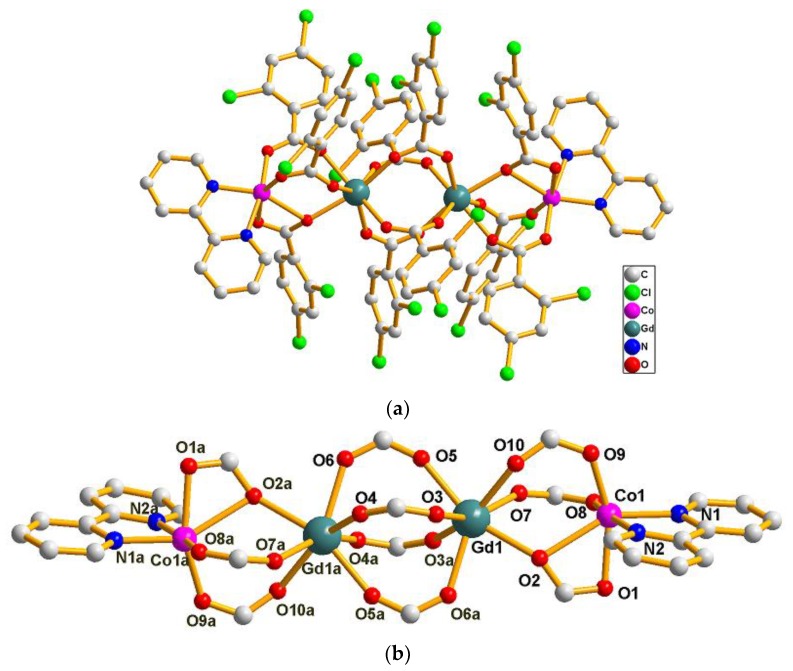
(**a**) The molecule structure of the tetranuclear [Gd_2_Co_2_(L2)_10_(bipy)_2_] of **8**. Hydrogen atoms are omitted for clarity. (**b**) The coordination environments of the metal centers in **8**.

**Figure 4:**
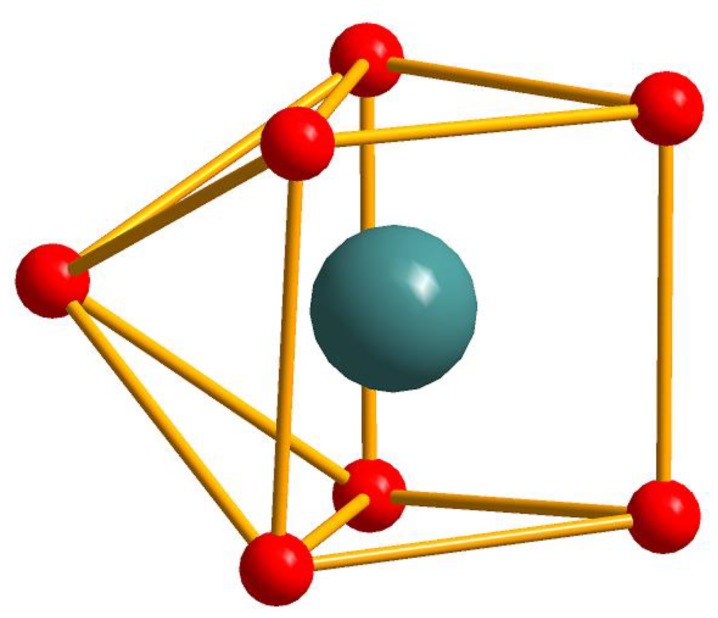
The distorted Capped Trigonal Prismatic coordination geometry of the Gd(III) ion in **8** with the GdO_7_ sphere.

**Figure 5:**
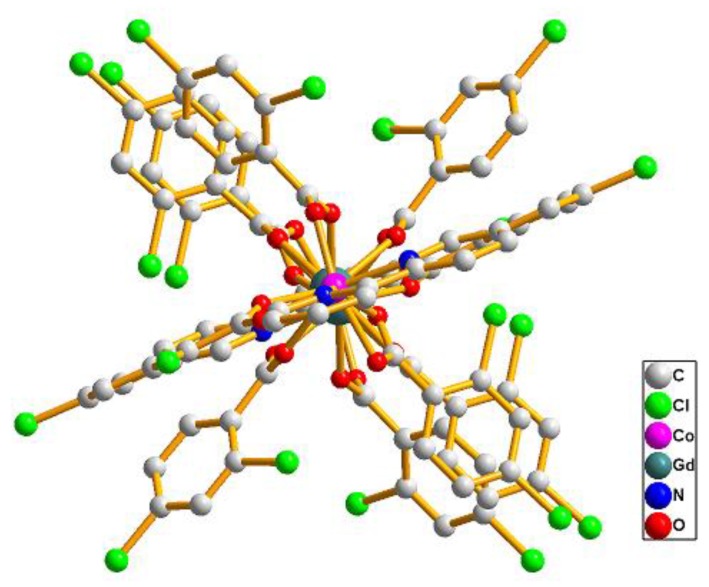
The paddlewheel arrangement of the ligands viewed along the Co1–Gd1–Gd1a–Co1a axis in **8**.

The single crystal X-ray analysis revealed that compounds **7**–**13** are isostructural. They crystallize in the triclinic *P*$\overset{-}{1}$ space group and consist of linear tetranuclear metal ions. Hence, only the structure of **8** is described in detail. As presented in Figure 3a, the structure of **8** contains two symmetry related Gd(III) ions (Gd1, Gd1a) (*a* = −*x*, −*y*, 1 − *z*), two symmetry related Co(II) ions (Co1 and Co1a) and ten symmetry related L2 ligands and two symmetry related bipy ligands. The coordination environment of the metal ions (Gd1, Gd1a, Co1 and Co1a) in **8** is presented in Figure 3b. Co1 is six-coordinated with distorted octahedron geometry, composed of four carboxylic O atoms (O1, O2, O8, O9) from three different L2 ligands and two N atoms (N1, N2) from one bipy ligand (Co1–O = 2.0096(19)–2.1374(19) Å, Co1–N = 2.073(2)–2.144(2) Å). The Gd1 ion is seven-coordinated with the distorted mono-capped trigonal prismatic coordination geometry, as depicted in Figure 4, and is composed of seven carboxylic O atoms from seven different L2 ligands (Gd1–O = 2.304(2)–2.3752(19) Å). Two bridging *μ-1,2* [bis(monodentate)] carboxylate groups and one *μ-1,1,2* [bidentate-monodentate] carboxylate group link the Gd1(III) ion and Co1(II) ion together (Gd1–Co1 = 4.0404(20) Å). Four bridging *μ-1,2* [bis(monodentate)] carboxylate groups link two symmetry related Gd1 and Gd1a together (Gd1-Gd1a = 4.3862(20) Å); two symmetry related Co(II) ions are located at the two sides of the two symmetry related Gd(III) ion (Co1–Co1a = 12.4559(59) Å, Co1–Gd1–Gd1a = 174.907(14)°). Co1–Gd1–Gd1a–Co1a is arranged almost linearly, and the paddlewheel type of ligand can be seen when the molecules are viewed along the Co1–Gd1–Gd1a–Co1a (*a* = −*x*, −*y*, 1 − *z*) axis (Figure 5). The two symmetry-related bipy ligands are located on the two ends as the terminal ligands.

#### 3.1.3. Crystal Structures of Compounds **14**–**16**

**Figure 6:**
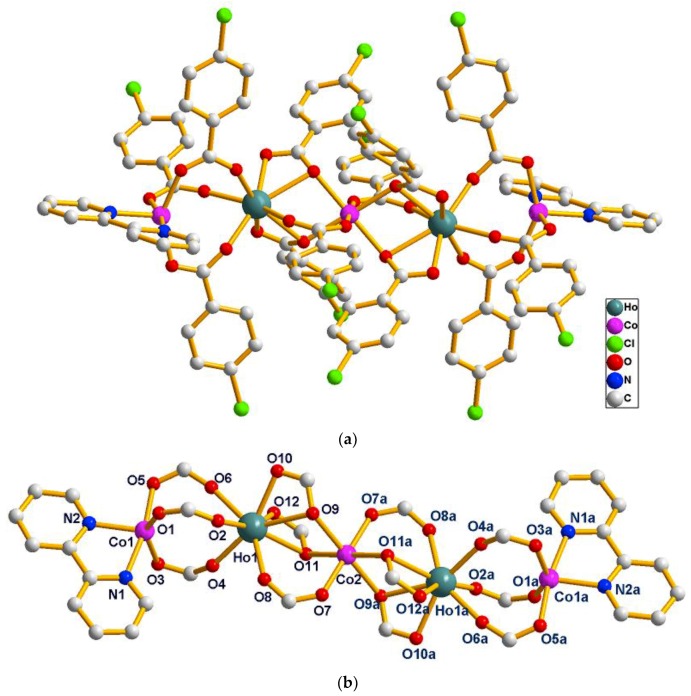
(**a**) The molecule structure of the pentanuclear [Ho_2_Co_3_(L1)_12_(bipy)_2_] of **14.** Hydrogen atoms are omitted for clarity. (**b**) The coordination environments of the metal centers in **14**.

**Figure 7:**
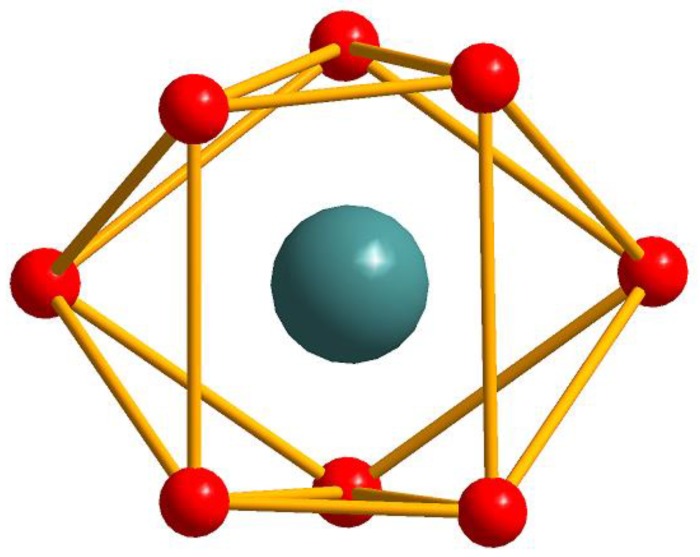
The distorted square antiprism coordination geometry of Ho(III) ion in **14** with the HoO_8_ sphere.

The single crystal X-ray analysis reveals that compounds **14**–**16** are isostructural. They crystallize in the triclinic *P*$\overset{-}{1}$ space group and consist of quasi-linear pentanuclear metal ions. Hence, only the structure of **14** is described in detail. As presented in Figure 6a, the asymmetric unit of **14** contains two symmetry related Ho(III) ions (Ho1, Ho1a), three Co(II) ions (Co1, Co1a and Co2) and twelve symmetry related L1 ligands and two symmetry related bipy ligands. The coordination environment of the metal ions (Ho1, Ho1a, Co1, Co1a and Co2) in **14** is presented in Figure 6b. The Co2(II) ion is located on the crystallographic inversion center, which is six-coordinated with distorted octahedron geometry, composed of six carboxylic O atoms (O7, O7a, O9, O9a, O11, O11a) from six different L1 ligands (Co2–O = 2.010(3)–2.167(3) Å). The Ho1 ion is eight-coordinated with a distorted square anti-prism coordination geometry, as depicted in Figure 7, and is composed of eight carboxylic O atoms from six different L1 ligands (Ho1–O = 2.239(4)–2.580(3) Å). One bridging *μ-1,2* [bis(monodentate)] carboxylate group and two *μ-1,1,2* [bidentate-monodentate] carboxylate groups link the Ho1(III) ion and Co2(II) ion together (Ho–Co2 = 3.6673(17) Å, Ho1–Co2–Ho1a = 180.00°). Co1 is six-coordinated with distorted octahedron geometry, and is composed of three carboxylic O atoms (O1, O3, O5) from three different L1 ligands and two N atoms (N1, N2) from one bipy ligand (Co1–O = 2.010(3)–2.051(3) Å, Co1–N = 2.077(4)–2.142(4) Å). The three bridging *μ-1,2* [bis(monodentate)] carboxylate group links Ho1 and Co1 together (Ho1–Co1 = 4.0210(22) Å, Co1–Ho1–Co2 = 162.073(18)°). Co1–Ho1–Co2–Ho1a–Co1a is arranged almost linearly; the paddlewheel type of ligand can be seen when the molecules are viewed along the Co1–Ho1–Co2–Ho1a–Co1a (*a* = 2 − *x*, 1 − *y*, 1 − *z*) axis (Figure S1). The two symmetry related bipy ligands are located on the two ends as the terminal ligands. The pentanuclear compounds reported in the literature are mostly concentrated in the 3d metal region [23,24,25,26], and the pentanuclear compounds of **3**d–**4**f heterometallic metals are relatively few [27,28,29]. In the reported **3**d–**4**f pentanuclear compounds, the arrangement shapes of pentanuclear metals can be roughly divided into the following types: (a) Trigonal-bipyramidal type [30], (b) Goblet-like [31], (c) Butterfly-shaped [32], (d) Open-book type [33], (e) T-shaped [34] and (f) Sandwich-type [35]. Only one case is similar to the linear structure reported in this paper [36].

The differences between the three types of structures may be ascribed to the stereo-hindrance effect of the carboxylate ligands and the different radii of Ln(III) ions.

### 3.2. Magnetic Properties

Magnetic susceptibility measurements were carried out on polycrystalline samples of **3**–**18** in the temperature range of 2–300 K at 1000 Oe. The basic magnetic data derived from these measurements are listed in Table 6. The small discrepancies between the calculated and theoretical values of χ_m_T might be ascribed to the orbital contribution of the metal ion. This phenomenon is quite common in the literature [37,38,39,40,41]. Their calculated χ_m_T values are also close to those of compounds with similar structures [42].

#### 3.2.1. Magnetic Properties of Compounds **3**–**6**

**Figure 8:**
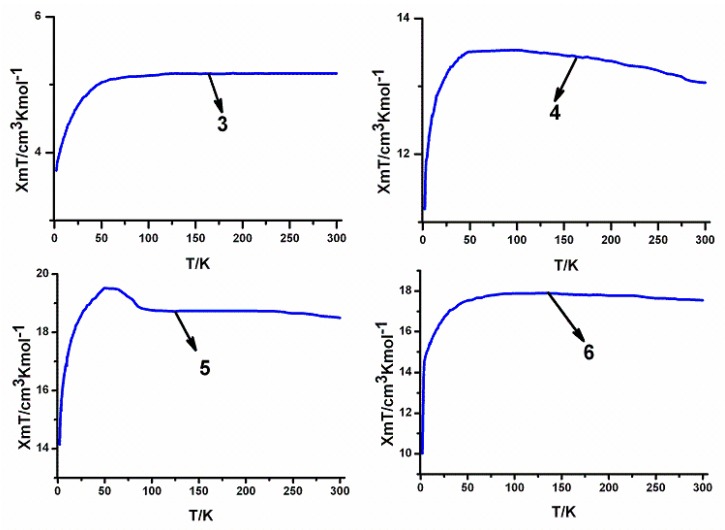
Temperature dependence of the χ_m_T curves at 1000 Oe for the series of heterotrinuclear compounds **3**–**6**.

The experimental χ_m_T values at 300 K for the series of heterotrinuclear compounds **3**–**6** are 5.16, 13.05, 18.52 and 17.54 cm^3^·K·mol^−1^, which are close to those expected for two uncoupled Co(II) ions (S = 3/2, g = 2) and one lanthanide metal ion: one Sm(III) (*g*_J_ = 2/7, ^6^H_5/2_) for **3**, one Gd(III) (*g*_J_ = 2, ^8^S_7/2_) for **4**, one Dy(III) (*g*_J_ = 4/3, ^6^H_15/2_) for **5**, one Tb(III) (*g*_J_ = 3/2, ^7^F_6_) for **6**. As shown in Figure 8, the magnetic properties of the four compounds are relatively different when studied as a function of the temperature. When the temperature is decreased, the χ_m_T value of **3** at 1000 Oe progressively decreases to reach 3.53 cm^3^·K·mol^−1^ at 2 K. In this situation, it is very difficult to interpret the magnetic properties of **3**, because Sm(III) ions of this system have an orbital degenerate ground state which can cause the same feature.^23^ On the other hand, the χ_m_T values of **4**, **5** and **6** at 1000 Oe continuously increase upon lowering the temperature to reach 13.53, 19.52 and17.90 cm^3^·K·mol^−1^, respectively, at 94 K, 50 K and 124 K. For **4**, the Gd(III) ion, whose f-f spin-orbit coupling effect is absent in the first order (for the ^8^S_7/2_ ground state), the influence of the ligand field can be safely neglected, and the increase of χ_m_T value can be ascribed to the ferromagnetic interactions in this trinuclear compounds. For **5** and **6**, the ground states of the Dy(III) and Tb(III) ions are ^6^H_15/2_ and ^7^F_6_, respectively. Upon lowering the temperature, progressive depopulation of these levels occurs, and the increase of these χ_m_T values may be ascribed to the ferromagnetic interactions among Dy(III)/Tb(III) and Co(II) ions in these trinuclear compounds [37]. At lower temperatures, χ_m_T decreases in these cases. This is probably the result of magnetic anisotropy or weak antiferromagnetic interactions between trinuclear compounds.

#### 3.2.2. Magnetic Properties of Compounds **7**–**13**

**Figure 9:**
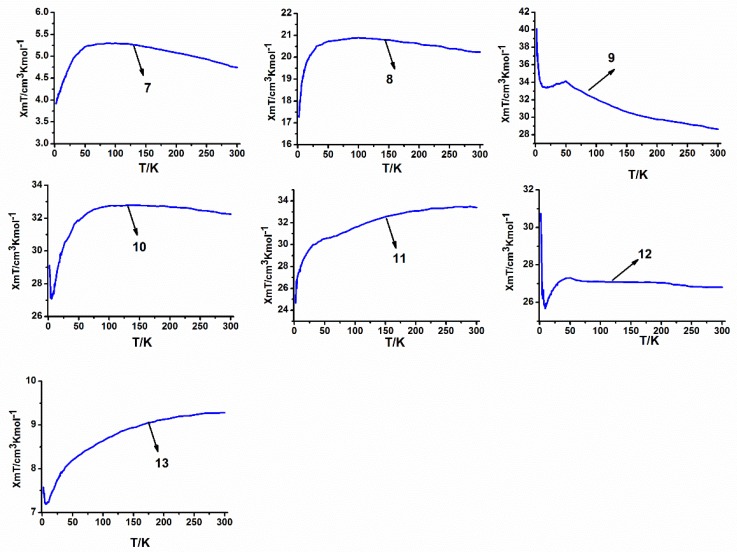
Temperature dependence of the χ_m_T curves at 1000 Oe for the series of heterotetranuclear compounds **7**–**13.**

The plots of χ_m_T vs T for the heterotetranuclear compounds **7**–**13** are presented in Figure 9. The experimental χ_m_T values at room temperature are 4.74, 20.23, 28.76, 32.24, 33.40, 26.82 and 9.28 cm^3^·K·mol^−1^, which are close to those expected for noninteracting metal ions of two Co(II) ions (*S* = 3/2, *g* = 2) and two lanthanide metal ions [3.93, 19.51, 27.39, 32.09, 31.89, 27.71 and 8.89 cm^3^·K·mol^−1^ for **7**–**13** respectively]. As shown in Figure 9, the magnetic properties of the eight compounds are relatively different when studied as a function of the temperature. When the temperature is decreased, the χ_m_T values of **7**, **9**, **10** and **12** at 1000 Oe continuously increase upon lowering the temperature to reach 5.30, 20.89, 34.10, 32.80 and 27.30 cm^3^·K·mol^−1^, respectively, at 89 K, 99 K, 48.9 K, 134 K and 48.9 K. For **8,** the Gd(III) ion, whose f-f spin-orbit coupling effect is absent in the first order (for the ^8^S_7/2_ ground state), the influence of the ligand field can be safely neglected, and the increase of χ_m_T value can be ascribed to the ferromagnetic interactions in the these tetranuclear compounds. For **7**, **9**, **10** and **12**, the ground states of the Sm(III), Tb(III), Dy(III) and Er(III) ions are ^6^H_5/2_, ^7^F_6_, ^6^H_15/2_ and ^4^I_15/2_, respectively. Upon lowering the temperature, progressive depopulation of these levels occurs, and the increase of these χ_m_T values may be ascribed to the ferromagnetic interactions among Ln(III) (Ln = Sm, Dy, Tb, Er) and Co(II) ions in these tetranuclear compounds [37]. At lower temperatures, χ_m_T decreases in these cases, probably as the result of magnetic anisotropy or weak antiferromagnetic interactions between tetranuclear compounds. On the other hand, when the temperature is decreased, the χ_m_T values of **11** and **13** at 1000 Oe progressively decrease to reach 24.68 and 7.18 cm^3^·K·mol^−1^, respectively, at 2 K and 5.9 K. In this situation, it is very difficult to interpret the magnetic properties of **11** and **13**, because the Ho(III) and Yb(III) ions of these systems have an orbital degenerate ground state which can cause the same feature.

We also synthesized the corresponding Y(III)–Co(II) heterometallic compounds (compound of **17** trinuclear and tetranuclear compound of **18**) to do the accurate diamagnetic correction with the 4-chlorobenzoic acid and 2,4-dichlorobenzoic acid as ligands, respectively [43]. The crystal data of compounds **17** and **18** are listed in Table S4 in the Supporting Information. The plots of χ_m_T vs T for **17** and **18** are shown in Figures S2 and S3 in the Supporting Information. The comparison between the isostructural Ln–Co and Y–Co compounds, which is defined as the function Δ(χ_m_T) = (χ_m_T)_Ln-Co_ − (χ_m_T)_Y-Co_, may eliminate the crystal field contribution of Co ions. Thus, according to the change trend of Δ(χ_m_T) in the 2–300 K temperature range (Figures S4 and S5 in the Supporting Information), the ferromagnetic or antiferromagnetic coupling of Ln^3+^ would be clearly concluded. However, it is difficult to comment on the interactions of Co–Ln in these compounds, with the Co^2+^ and Ln^3+^ ions both having intrinsic complicated magnetic characteristics which include the presence of spin–orbit coupling and magnetic anisotropy.

#### 3.2.3. Magnetic Properties of Compounds **14**–**16**

**Figure 10:**
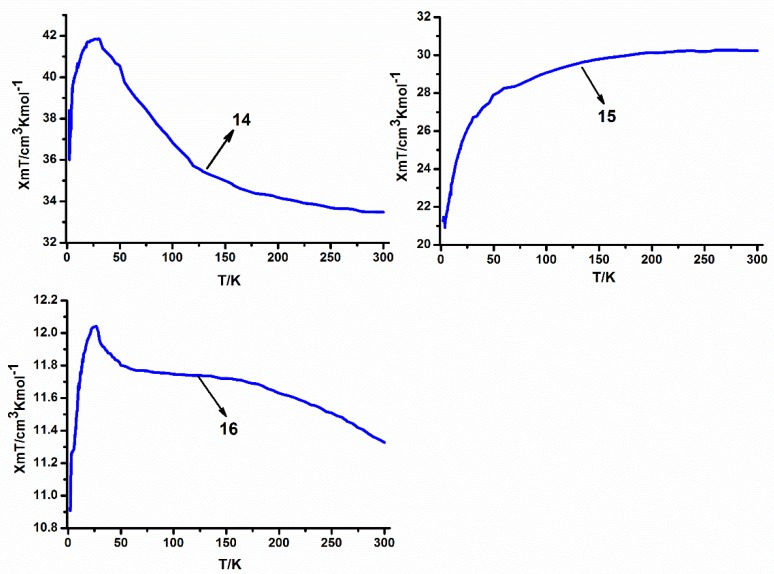
Temperature dependence of the χ_m_T curves at 1000 Oe for the series of heteropentanuclear compounds **14**–**16**.

The experimental χ_m_T values at room temperature for the series of heteropentanuclear compounds [33.78, 30.23 and 11.33 cm^3^·K·mol^−1^ for **14**–**16**, respectively] are close to those expected for noninteracting metal ions of the three Co(II) ions (*S* = 3/2, *g* = 2) and two lanthanide metal ions [33.77 cm^3^·K·mol^−1^ for **14** (Ho), 28.59 cm^3^·K·mol^−1^ for **15** (Er) and 10.76 for **16** (Yb)]. As shown in Figure 10, the magnetic properties of the three compounds are relatively different when studied as a function of the temperature. When the temperature is decreased, the χ_m_T values of **14** and **16** at 1000 Oe continuously increase upon lowering the temperature to reach 41.78 and 12.04m^3^·K·mol^−1^, respectively, at 22.9 K and 24.9 K. For **14**, and **16**, the ground states of the Ho(III) and Yb(III) ions are ^5^I_8_ and ^2^F_7/2_, respectively. Upon lowering the temperature, progressive depopulation of these levels occurs, and the increase of these χ_m_T value may be ascribed to the ferromagnetic interactions among Ho(III), Yb(III) and Co(II) ions in these pentanuclear compounds. At lower temperatures, χ_m_T decreases in these cases, probably as the result of magnetic anisotropy or weak antiferromagnetic interactions between pentanuclear compounds. On the other hand, when the temperature is decreased, the χ_m_T value of **15** at 1000 Oe progressively decreases to reach 21.27cm^3^·K·mol^−1^ at 2 K. In this situation, it is very difficult to interpret the magnetic properties of **15**, because Er(III) ions of this system have an orbital degenerate ground state which can cause the same feature [37].

To probe the presence of slow dynamics in these molecular systems and thus the presence of SMM behaviour, ac susceptibility measurements were performed systematically for compounds **5**, **9** and **14**. The measurements were collected at zero direct-current (dc) field with an ac field of 3 Oe. Unfortunately, only compound **14** displayed nonzero and rather smaller frequency-dependent out-of-phase signals (Figures S6 and S7 in the Supporting Information). Even so, this phenomenon should not be neglected, because the frequency-dependent out-of-phase signals often occur in Dy(III)-containing systems and are often suppressed by applying a static dc field [37], which is rarely reported in Ho(III)-containing compounds in a zero direct-current (dc) field.

## 4. Conclusions

In summary, eighteen **3**d–**4**f heterometallic coordination compounds were successfully synthesized under hydrothermal conditions. Structural analyses revealed that the eighteen compounds including three types, the compounds **1**–**6** and **17**, are isostructural heterotrinuclear compounds, the compounds **7**–**13** and **18** are isostructural heterotetranuclear compounds and **14**–**16** are isostructural heteropentanuclear compounds. It is noteworthy that compound **14**, which contains Ho(III) ions, presented field-induced slow magnetic relaxation. The successful preparation of these series of compounds provides valuable information for further construction of more **3**d–**4**f materials with novel magnetic properties.

## Figures and Tables

**Table 1:** Crystal data and structure refinement information for **1**–**3**.

Compound	1	2	3
Empirical formula	C_69_H_44_Cl_7_Co_2_N_4_O_14_Pr	C_69_H_44_Cl_7_Co_2_N_4_O_14_Eu	C_69_H_44_Cl_7_Co_2_N_4_O_14_Sm
Fw	1660.00	1671.05	1669.45
Temperature (K)	293(2)	293(2)	293(2)
Crystal system	Triclinic	Triclinic	Triclinic
Space group	*P*$\overset{-}{1}$	*P*$\overset{-}{1}$	*P*$\overset{-}{1}$
a(Å)	11.476(2)	11.545(2)	11.734(2)
b(Å)	13.762(3)	13.651(3)	13.617(3)
c(Å)	21.363(4)	21.169(4)	21.542(4)
α (°)	80.87(3)	99.16(3)	80.12(3)
β (°)	89.82(3)	90.04(3)	89.88(3)
γ (°)	89.18(3)	90.98(3)	89.99(3)
V (Å^3^)	3330.8(11)	3293.2(11)	3391.0(11)
Z	2	2	2
F(000)	1660	1668	1666
*D* (Mg/m^3^)	1.655	1.685	1.635
Abs coeff (mm^−1^)	1.562	1.793	1.682
Data/restraints/params	15,554/0/874	15,461/0/874	15,927/486/920
GOF	0.999	1.004	1.031
R_1_^a^ (I = 2σ(I))	0.0734	0.0398	0.0582
WR_2_^a^ (all data)	0.1669	0.1004	0.1373

^a^ R_1_ = Σ||F_o_| − |F_c_||/|F_o_|, wR_2_ = [Σw(F_o_^2^ − F_c_^2^)^2^/Σw(F_o_^2^)^2^]^1/2^.

**Table 2:** Crystal data and structure refinement information for **4**–**6.**

Compound	4	5	6
Empirical formula	C_69_H_44_Cl_7_Co_2_N_4_O_14_Gd	C_69_H_44_Cl_7_Co_2_N_4_O_14_Tb	C_69_H_44_Cl_7_Co_2_N_4_O_14_Dy
Fw	1676.34	1678.02	1681.59
Temperature (K)	293(2)	293(2)	293(2)
Crystal system	Triclinic	Triclinic	Triclinic
Space group	*P*$\overset{-}{1}$	*P*$\overset{-}{1}$	*P*$\overset{-}{1}$
a(Å)	11.769(2)	11.783(2)	11.608(2)
b(Å)	13.587(3)	13.584(3)	13.596(3)
c(Å)	21.539(4)	21.484(4)	21.107(4)
α (°)	80.09(3)	80.06(3)	99.23(3)
β (°)	89.73(3)	89.77(3)	90.12(3)
γ (°)	89.97(3)	89.95(3)	90.94(3)
V (Å^3^)	3392.8(11)	3387.1(11)	3287.5(11)
Z	2	2	2
F(000)	1670	1672	1674
D (Mg/m^3^)	1.641	1.645	1.699
Abs coeff (mm^−1^)	1.793	1.861	1.978
Data/restraints/params	11,687/1512/920	15,502/0/920	15,419/0/874
GOF	1.008	1.039	0.979
R_1_^a^ (I = 2σ(I))	0.0785	0.0429	0.0461
WR_2_^a^ (all data)	0.1684	0.0914	0.1001

^a^ R_1_ = Σ||F_o_| − |F_c_||/|F_o_|, wR_2_ = [Σw(F_o_^2^ − F_c_^2^)^2^/Σw(F_o_^2^)^2^]^1/2^.

**Table 3:** Crystal data and structure refinement information for **7–10.**

Compound	7	8	9	10
Empirical formula	C_90_H_46_Cl_20_Co_2_Sm_2_N_4_O_20_	C_90_H_46_Cl_20_Co_2_Gd_2_N_4_O_20_	C_90_H_46_Cl_20_Co_2_Tb_2_N_4_O_20_	C_90_H_46_Cl_20_Co_2_Dy_2_N_4_O_20_
Fw	2630.87	2644.67	2648.01	2655.17
Temperature (K)	293(2)	293(2)	293(2)	293(2)
Crystal syst.	Triclinic	Triclinic	Triclinic	Triclinic
Space group	*P*$\overset{-}{1}$	*P*$\overset{-}{1}$	*P*$\overset{-}{1}$	*P*$\overset{-}{1}$
a(Å)	12.998(3)	13.411(3)	13.421(3)	13.048(3)
b(Å)	13.914(3)	13.804(3)	13.786(3)	13.774(3)
c(Å)	14.241(3)	13.809(3)	13.806(3)	13.809(3)
α (°)	101.41(3)	98.96(3)	99.04(3)	99.20(3)
β (°)	97.92(3)	107.45(3)	95.86(3)	95.81(3)
γ (°)	106.52(3)	95.93(3)	107.45(3)	107.60(3)
V (Å^3^)	2368.3(9)	2378.3(9)	2376.2(9)	2305.5(9)
Z	1	1	1	1
F(000)	1292	1296	1298	1300
*D* (Mg/m^3^)	1.845	1.847	1.850	1.912
Abs coeff (mm^−1^)	2.203	2.353	2.448	2.610
Data/restraints/params	11,077/0/623	11,128/0/623	11,077/0/623	11,038/0/623
GOF	0.953	1.014	0.953	0.886
R_1_^a^ (I = 2σ(I))	0.0326	0.0294	0.0326	0.0274
WR_2_^a^ (all data)	0.0704	0.0634	0.0704	0.0668

^a^ R_1_ = Σ||F_o_| − |F_c_||/|F_o_|, wR_2_ = [Σw(F_o_^2^ − F_c_^2^)^2^/Σw(F_o_^2^)^2^]^1/2^.

**Table 4:** Crystal data and structure refinement information for **11**–**13.**

Compound	11	12	13
Empirical formula	C_90_H_46_Cl_20_Co_2_Ho_2_N_4_O_20_	C_90_H_46_Cl_20_Co_2_Er_2_N_4_O_20_	C_90_H_46_Cl_20_Co_2_Yb_2_N_4_O_20_
Fw	2660.03	2664.69	2676.25
Temperature (K)	293(2)	293(2)	293(2)
Crystal syst.	Triclinic	Triclinic	Triclinic
Space group	*P*$\overset{-}{1}$	*P*$\overset{-}{1}$	*P*$\overset{-}{1}$
a(Å)	13.425(3)	13.397(3)	13.503(3)
b(Å)	13.774(3)	13.741(3)	13.801(3)
c(Å)	13.821(3)	13.802(3)	13.877(3)
α (°)	99.27(3)	99.44(3)	99.98(3)
β (°)	95.79(3)	95.77(3)	95.65(3)
γ (°)	107.66(3)	107.65(3)	107.71(3)
V (Å^3^)	2372.8(9)	2357.7(9)	2394.5(9)
Z	1	1	1
F(000)	1302	1304	1308
D (Mg/m^3^)	1.862	1.877	1.856
Abs coeff (mm^−1^)	2.629	2.747	2.905
Data/restraints/params	11,021/0/623	11,051/0/623	10,813/0/622
GOF	0.993	0.987	1.036
R_1_^a^ (I = 2σ(I))	0.0402	0.0317	0.0379
WR_2_^a^ (all data)	0.0908	0.0710	0.0874

^a^ R_1_ = Σ||F_o_| − |F_c_||/|F_o_|, wR_2_ = [Σw(F_o_^2^ − F_c_^2^)^2^/Σw(F_o_^2^)^2^]^1/2^.

**Table 5:** Crystal data and structure refinement information for **14**–**16.**

Compound	14	15	16
Empirical formula	C_104_H_64_Cl_12_Co_3_Ho_2_N_4_O_24_	C_104_H_64_Cl_12_Co_3_Er_2_N_4_O_24_	C_104_H_64_Cl_12_Co_3_Yb_2_N_4_O_24_
Fw	2685.64	2690.30	2701.86
Temperature (K)	293(2)	293(2)	293(2)
Crystal syst.	Triclinic	Triclinic	Triclinic
Space group	*P*$\overset{-}{1}$	*P*$\overset{-}{1}$	*P*$\overset{-}{1}$
a(Å)	13.900(3)	13.893(3)	13.702(3)
b(Å)	14.772(3)	14.824(3)	14.600(3)
c(Å)	16.034(3)	16.073(3)	15.895(3)
α (°)	105.01(3)	105.25(3)	105.13(3)
β (°)	103.74(3)	103.61(3)	103.46(3)
γ (°)	113.66(3)	113.49(3)	113.30(3)
V (Å^3^)	2687.0(16)	2703.9(9)	2608.9(9)
Z	1	1	1
F(000)	1327	1329	1333
D (Mg/m^3^)	2.280	1.652	1.720
Abs coeff (mm^−1^)	2.448	2.354	2.624
data/restraints/params	12,585/0/683	12,603/0/683	12,324/6/683
GOF	1.001	0.998	1.006
R1a (I = 2σ(I))	0.0500	0.0461	0.0468
WR2a (all data)	0.1127	0.1111	0.1056

^a^ R_1_ = Σ||F_o_| − |F_c_||/|F_o_|, wR_2_ = [Σw(F_o_^2^ − F_c_^2^)^2^/Σw(F_o_^2^)^2^]^1/2^.

**Table 6:** Summary of static magnetic properties of the three series of heterometallic compounds **3**–**14**.

	**3** (SmCo_2_)	**4** (GdCo_2_)	**5** (DyCo_2_)	**6** (TbCo_2_)	**7** (Sm_2_Co_2_)
Ground state of Ln(III)	^6^H_5/2_	^8^S_7/2_	^6^H_15/2_	^7^F_6_	^6^H_5/2_
χ_m_T (theoretical)	3.84	11.63	17.92	15.57	3.93
χ_m_T (observed)	5.16	13.05	18.52	17.54	4.74
C (emu mol^−1^ K)	5.16	12.83	18.48	17.38	4.32
*θ* (K)	−0.33	7.05	2.03	3.89	29.09
	**8** (Gd_2_Co_2_)	**9** (Tb_2_Co_2_)	**10** (Dy_2_Co_2_)	**11** (Ho_2_Co_2_)	**12** (Er_2_Co_2_)
Ground state of Ln(III)	^8^S_7/2_	^7^F_6_	^6^H_15/2_	^5^I_8_	^4^I_15/2_
χ_m_T (theoretical)	19.51	27.39	32.09	31.89	26.71
χ_m_T (observed)	20.23	28.76	32.24	33.40	26.82
C (emu mol^−1^ K)	19.81	27.38	32.18	34.58	26.66
*θ* (K)	7.14	15.49	2.36	−9.29	2.11
	**13** (Yb_2_Co_2_)	**14** (Ho_2_Co_3_)	**15** (Er_2_Co_3_)	**16** (Yb_2_Co_3_)	
Ground state of Ln(III)	^2^F_7/2_	^5^I_8_	^4^I_15/2_	^2^F_7/2_	
χ_m_T (theoretical)	8.89	33.77	28.59	10.76	
χ_m_T (observed)	9.28	33.78	30.23	11.33	
C (emu mol^−1^ K)	9.66	33.04	30.97	11.11	
*θ* (K)	−11.87	12.54	−6.23	8.01	

## Supplementary Materials

The following are available online at http://www.mdpi.com/2073-4360/11/2/196/s1, CCDC-1887007 (for **1**), 1887008 (for **2**), 1887009 (for **3**), 1887010 (for **4**) 1887011 (for **5**), 1887012 (for **6**), 1887013 (for **7**), 1887014 (for **8**), CCDC-1887015 (for **9**), 1887016 (for **10**), 1887278 (for **11**) and 1887279 (for **12**), 1887280 (for **13**), 1887501 (for **14**), 1887500 (for **15**) 1887502 (for **16**), 1887282 (for **17**), and 1887283 (for **18**) contain the supplementary crystallographic data for this paper. These data can be obtained free of charge from The Cambridge Crystallographic Data Centre via www.ccdc.cam.ac.uk/data_request/cif.
