# Supplementary material for: Syntheses, Structures, and Magnetic Properties of a Series of Heterotri-, Tetra- and Pentanuclear LnIII–CoII Compounds
Source: Polymers (Basel). 2019 Jan 23;11(2):196. doi: 10.3390/polym11020196 (PMC6418803; doi:10.3390/polym11020196)
Supplement: Supplementary file 1 [file polymers-11-00196-s001.pdf]

Supporting information

Table S1. Selected bond distances (Å) and angles (°) for compounds **1**–**6**.

	**1**		
Pr(1)-O(14)	2.412(4)	Pr(1)-O(7)	2.495(5)
Pr(1)-O(2)	2.419(4)	Pr(1)-O(8)	2.531(4)
Pr(1)-O(10)	2.438(4)	Co(2)-O(9)	2.038(4)
Pr(1)-O(12)	2.445(4)	Co(2)-O(13)	2.040(4)
Pr(1)-O(5)	2.457(4)	Co(2)-N(4)	2.096(5)
Pr(1)-O(3)	2.458(4)	Co(2)-O(12)	2.139(4)
Co(2)-N(3)	2.154(5)	Co(1)-N(2)	2.133(5)
Co(1)-O(6)	1.986(5)	Co(1)-O(3)	2.198(4)
Co(1)-O(1)	2.094(4)	Co(1)-O(4)	2.314(5)
Co(1)-N(1)	2.105(5)	O(14)-Pr(1)-O(2)	140.99(15)
O(14)-Pr(1)-O(10)	71.61(15)	O(14)-Pr(1)-O(5)	142.94(16)
O(2)-Pr(1)-O(10)	72.58(15)	O(2)-Pr(1)-O(5)	74.87(15)
O(10)-Pr(1)-O(12)	80.83(14)	O(14)-Pr(1)-O(3)	111.92(14)
O(2)-Pr(1)-O(3)	76.56(14)	O(2)-Pr(1)-O(7)	130.55(14)
O(10)-Pr(1)-O(3)	80.62(14)	O(10)-Pr(1)-O(7)	140.47(14)
O(12)-Pr(1)-O(3)	155.45(15)	O(12)-Pr(1)-O(7)	72.96(14)
O(12)-Pr(1)-O(8)	122.96(14)	O(2)-Pr(1)-O(8)	144.36(14)
O(5)-Pr(1)-O(8)	73.95(14)	O(9)-Co(2)-O(13)	95.66(17)
O(3)-Pr(1)-O(8)	81.50(14)	O(6)-Co(1)-O(3)	101.53(18)
O(7)-Pr(1)-O(8)	52.50(13)	O(1)-Co(1)-O(3)	111.04(16)
O(13)-Co(2)-O(12)	108.98(17)	O(9)-Co(2)-O(12)	99.55(16)
O(6)-Co(1)-O(4)	84.3(2)	O(3)-Co(1)-O(4)	58.35(16)
O(1)-Co(1)-O(4)	167.44(16)	O(6)-Co(1)-O(1)	91.96(19)
	**2**		
Eu(1)-O(9)	2.360(2)	Eu(1)-O(12)	2.375(2)
Eu(1)-O(4)	2.368(2)	Eu(1)-O(5)	2.382(2)
Eu(1)-O(14)	2.402(2)	Co(1)-O(1)	1.981(3)
Eu(1)-O(2)	2.403(2)	Co(1)-O(3)	2.080(2)
Eu(1)-O(7)	2.429(2)	Co(1)-N(1)	2.097(3)
Eu(1)-O(8)	2.488(2)	Co(1)-N(2)	2.127(3)
Co(1)-O(5)	2.225(2)	Co(2)-O(10)	2.034(2)
Co(1)-O(6)	2.263(3)	Co(2)-N(4)	2.081(3)
Co(2)-O(13)	2.024(2)	Co(2)-O(12)	2.141(2)
Co(2)-N(3)	2.151(3)	Co(2)-O(11)	2.380(2)
O(9)-Eu(1)-O(4)	141.12(8)	O(4)-Eu(1)-O(5)	78.47(8)
O(9)-Eu(1)-O(12)	79.55(8)	O(12)-Eu(1)-O(5)	155.50(8)
O(4)-Eu(1)-O(12)	80.90(8)	O(9)-Eu(1)-O(14)	71.98(8)
O(9)-Eu(1)-O(5)	109.07(8)	O(4)-Eu(1)-O(14)	72.14(8)
O(12)-Eu(1)-O(14)	81.61(8)	O(12)-Eu(1)-O(2)	105.59(8)

O(5)-Eu(1)-O(14)	79.62(8)	O(5)-Eu(1)-O(2)	81.63(8)
O(9)-Eu(1)-O(2)	142.94(8)	O(14)-Eu(1)-O(2)	144.70(8)
O(4)-Eu(1)-O(2)	74.97(8)	O(9)-Eu(1)-O(7)	74.13(8)
O(4)-Eu(1)-O(7)	130.24(8)	O(4)-Eu(1)-O(8)	144.27(8)
O(12)-Eu(1)-O(7)	72.74(8)	O(12)-Eu(1)-O(8)	124.20(7)
O(5)-Eu(1)-O(7)	131.31(8)	O(5)-Eu(1)-O(8)	80.22(8)
O(9)-Eu(1)-O(8)	73.38(8)	O(7)-Eu(1)-O(8)	53.39(7)
O(13)-Co(2)-O(12)	98.75(9)	O(1)-Co(1)-O(5)	100.96(10)
O(10)-Co(2)-O(12)	107.58(9)	O(3)-Co(1)-O(5)	110.27(9)
O(13)-Co(2)-O(11)	96.50(9)	O(1)-Co(1)-O(6)	84.48(11)
O(13)-Co(2)-O(10)	94.89(9)	O(3)-Co(1)-O(6)	167.15(8)
		**3**	
Sm(1)-O(12)	2.377(3)	Sm(1)-O(4)	2.398(3)
Sm(1)-O(2)	2.378(3)	Sm(1)-O(14)	2.443(3)
Sm(1)-O(8)	2.384(4)	Sm(1)-O(13)	2.512(3)
Sm(1)-O(10)	2.395(4)	Co(1)-O(3)	2.028(3)
Sm(1)-O(5)	2.396(3)	Co(1)-O(1)	2.036(3)
Co(1)-N(1)	2.079(4)	Co(2)-O(7)	2.070(4)
Co(1)-O(5)	2.153(3)	Co(2)-N(4)	2.098(4)
Co(1)-N(2)	2.153(4)	Co(2)-N(3)	2.123(4)
Co(1)-O(6)	2.399(4)	Co(2)-O(11)	2.187(5)
Co(2)-O(9)	1.982(4)	Co(2)-O(12)	2.359(4)
O(12)-Sm(1)-O(8)	78.48(13)	O(2)-Sm(1)-O(10)	143.38(13)
O(2)-Sm(1)-O(8)	141.78(13)	O(8)-Sm(1)-O(10)	74.39(14)
O(8)-Sm(1)-O(5)	82.03(12)	O(12)-Sm(1)-O(5)	155.72(12)
O(10)-Sm(1)-O(5)	107.14(13)	O(2)-Sm(1)-O(5)	79.98(12)
O(12)-Sm(1)-O(4)	80.06(13)	O(5)-Sm(1)-O(4)	80.25(12)
O(2)-Sm(1)-O(4)	71.85(13)	O(12)-Sm(1)-O(14)	131.69(12)
O(10)-Sm(1)-O(14)	73.84(13)	O(8)-Sm(1)-O(13)	143.94(12)
O(5)-Sm(1)-O(14)	72.42(11)	O(10)-Sm(1)-O(13)	73.57(13)
O(4)-Sm(1)-O(14)	139.59(12)	O(5)-Sm(1)-O(13)	123.24(11)
O(12)-Sm(1)-O(13)	80.80(12)	O(4)-Sm(1)-O(13)	132.36(14)
O(3)-Co(1)-O(1)	95.13(15)	O(9)-Co(2)-O(7)	91.39(18)
O(7)-Co(2)-O(12)	109.37(14)	O(7)-Co(2)-O(11)	165.62(14)
O(11)-Co(2)-O(12)	57.12(14)	O(9)-Co(2)-O(12)	101.87(16)
O(3)-Co(1)-O(5)	99.48(13)	O(9)-Co(2)-O(11)	87.2(2)
O(1)-Co(1)-O(5)	108.52(13)	O(5)-Co(1)-O(6)	57.47(12)
		**4**	
Gd(1)-O(10)	2.346(5)	Gd(1)-O(12)	2.387(5)
Gd(1)-O(4)	2.367(4)	Gd(1)-O(14)	2.420(6)
Gd(1)-O(2)	2.373(5)	Gd(1)-O(13)	2.489(6)
Gd(1)-O(7)	2.379(6)	Co(2)-O(11)	1.982(6)
Gd(1)-O(6)	2.383(6)	Co(2)-O(8)	2.073(6)
Co(2)-N(4)	2.092(6)	Co(1)-N(1)	2.075(6)

Co(2)-N(3)	2.124(7)	Co(1)-N(2)	2.146(6)
Co(2)-O(9)	2.164(6)	Co(1)-O(4)	2.166(5)
Co(1)-O(5)	2.017(5)	Co(1)-O(3)	2.378(6)
Co(1)-O(1)	2.029(5)	O(10)-Gd(1)-O(4)	155.9(2)
O(10)-Gd(1)-O(2)	104.89(19)	O(10)-Gd(1)-O(6)	79.31(19)
O(4)-Gd(1)-O(2)	81.17(17)	O(4)-Gd(1)-O(6)	80.70(18)
O(10)-Gd(1)-O(7)	79.94(19)	O(2)-Gd(1)-O(6)	71.6(2)
O(4)-Gd(1)-O(7)	81.11(18)	O(7)-Gd(1)-O(6)	71.5(2)
O(2)-Gd(1)-O(7)	141.1(2)	O(10)-Gd(1)-O(12)	82.67(19)
O(4)-Gd(1)-O(12)	106.38(17)	O(4)-Gd(1)-O(14)	72.33(18)
O(2)-Gd(1)-O(12)	143.9(2)	O(2)-Gd(1)-O(14)	74.98(19)
O(7)-Gd(1)-O(12)	74.6(2)	O(7)-Gd(1)-O(14)	130.58(18)
O(10)-Gd(1)-O(13)	80.09(19)	O(12)-Gd(1)-O(13)	73.47(19)
O(4)-Gd(1)-O(13)	123.67(17)	O(14)-Gd(1)-O(13)	53.03(16)
O(2)-Gd(1)-O(13)	73.26(19)	O(11)-Co(2)-O(8)	91.4(3)
O(7)-Gd(1)-O(13)	144.09(19)	O(5)-Co(1)-O(3)	97.7(2)
O(6)-Gd(1)-O(13)	132.62(18)	O(1)-Co(1)-O(3)	162.0(2)
O(11)-Co(2)-O(9)	87.5(3)	O(5)-Co(1)-O(4)	98.9(2)
O(8)-Co(2)-O(9)	165.3(2)	O(1)-Co(1)-O(4)	107.9(2)
	**5**		
Co(1)-O(1)	1.977(3)	Co(1)-O(8)	2.257(3)
Co(1)-O(11)	2.081(3)	Co(2)-O(10)	2.019(3)
Co(1)-N(1)	2.097(3)	Co(2)-O(6)	2.033(3)
Co(1)-N(2)	2.123(4)	Co(2)-N(4)	2.088(3)
Co(1)-O(7)	2.235(3)	Co(2)-O(13)	2.148(3)
Co(2)-N(3)	2.147(3)	Dy(1)-O(8)	2.331(2)
Co(2)-O(14)	2.354(3)	Dy(1)-O(12)	2.330(3)
Dy(1)-O(5)	2.321(3)	Dy(1)-O(13)	2.342(3)
Dy(1)-O(2)	2.360(3)	O(1)-Co(1)-O(11)	91.09(13)
Dy(1)-O(9)	2.368(3)	O(7)-Co(1)-O(8)	58.81(10)
Dy(1)-O(4)	2.403(3)	O(10)-Co(2)-O(6)	94.58(12)
Dy(1)-O(3)	2.469(3)	O(10)-Co(2)-O(14)	97.26(11)
O(1)-Co(1)-O(7)	85.08(13)	O(8)-Dy(1)-O(13)	155.90(10)
O(11)-Co(1)-O(7)	166.57(10)	O(12)-Dy(1)-O(13)	79.91(9)
O(5)-Dy(1)-O(9)	72.12(10)	O(2)-Dy(1)-O(9)	144.52(10)
O(8)-Dy(1)-O(4)	131.47(9)	O(5)-Dy(1)-O(4)	74.45(10)
O(1)-Co(1)-O(8)	100.50(12)	O(9)-Dy(1)-O(4)	140.36(9)
O(6)-Co(2)-O(13)	106.49(11)	O(5)-Dy(1)-O(3)	73.14(10)
O(13)-Co(2)-O(14)	58.10(10)	O(8)-Dy(1)-O(3)	79.53(10)
O(5)-Dy(1)-O(8)	107.11(9)	O(12)-Dy(1)-O(3)	144.35(9)
O(5)-Dy(1)-O(12)	141.20(10)	O(9)-Dy(1)-O(3)	131.30(10)
O(5)-Dy(1)-O(2)	142.86(10)	O(13)-Dy(1)-O(4)	72.24(9)
O(8)-Dy(1)-O(2)	82.34(9)	O(2)-Dy(1)-O(4)	72.90(10)
O(12)-Dy(1)-O(2)	75.17(10)	O(13)-Dy(1)-O(3)	124.51(9)

O(13)-Dy(1)-O(2)	104.73(9)	O(2)-Dy(1)-O(3)	73.55(10)
		**6**	
Tb(1)-O(12)	2.326(2)	Tb(1)-O(14)	2.410(2)
Tb(1)-O(4)	2.339(2)	Tb(1)-O(13)	2.497(2)
Tb(1)-O(6)	2.354(2)	Tb(1)-C(43)	2.797(3)
Tb(1)-O(10)	2.356(2)	Co(2)-O(7)	1.980(3)
Tb(1)-O(2)	2.362(3)	Co(2)-O(9)	2.059(3)
Tb(1)-O(8)	2.369(3)	Co(2)-N(4)	2.098(3)
Co(2)-N(3)	2.128(3)	Co(2)-O(12)	2.401(3)
Co(2)-O(11)	2.163(3)	Co(1)-O(1)	2.026(2)
Co(1)-O(3)	2.035(2)	Co(1)-N(2)	2.154(3)
Co(1)-N(1)	2.094(3)	Co(1)-O(6)	2.166(2)
Co(1)-O(5)	2.370(3)	O(12)-Tb(1)-O(4)	105.09(9)
O(12)-Tb(1)-O(6)	155.74(9)	O(6)-Tb(1)-O(10)	81.02(9)
O(4)-Tb(1)-O(6)	81.68(8)	O(12)-Tb(1)-O(2)	79.17(9)
O(12)-Tb(1)-O(10)	79.69(9)	O(4)-Tb(1)-O(2)	72.16(9)
O(4)-Tb(1)-O(10)	142.05(10)	O(6)-Tb(1)-O(2)	80.95(9)
O(10)-Tb(1)-O(2)	71.92(9)	O(10)-Tb(1)-O(8)	74.36(10)
O(12)-Tb(1)-O(8)	82.68(9)	O(2)-Tb(1)-O(8)	143.95(9)
O(4)-Tb(1)-O(8)	143.22(9)	O(12)-Tb(1)-O(14)	132.06(8)
O(6)-Tb(1)-O(8)	106.07(9)	O(4)-Tb(1)-O(14)	74.54(9)
O(2)-Tb(1)-O(14)	139.57(9)	O(10)-Tb(1)-O(13)	143.86(9)
O(14)-Tb(1)-O(13)	53.36(7)	O(12)-Tb(1)-O(4)	105.09(9)

Table S2**.** Selected bond distances (Å) and angles (°) for compounds **7**–**13**.

		**7**	
Co(1)-O(2)	2.023(2)	Co(1)-O(9)	2.368(2)
Co(1)-O(4)	2.0445(18)	Sm(1)-O(1)	2.3264(19)
Co(1)-N(2)	2.081(2)	Sm(1)-O(3)	2.3399(17)
Co(1)-N(1)	2.137(2)	Sm(1)-O(8)	2.3872(19)
Co(1)-O(10)	2.156(2)	Sm(1)-O(7)#1	2.4100(19)
Sm(1)-O(5)#1	2.4107(19)	Sm(1)-O(6)	2.4794(18)
Sm(1)-O(9)	2.416(2)	Sm(1)-O(5)	2.5430(19)
Sm(1)-Sm(1)#1	3.996(2)	O(7)-Sm(1)#1	2.4100(19)
O(5)-Sm(1)#1	2.4107(19)	O(2)-Co(1)-O(4)	92.96(8)
O(8)-Sm(1)-O(6)	96.34(8)	O(2)-Co(1)-O(10)	166.00(7)
O(7)#1-Sm(1)-O(6)	91.73(8)	O(4)-Co(1)-O(10)	93.19(8)
O(1)-Sm(1)-O(9)	75.85(7)	O(7)#1-Sm(1)-O(5)#1	71.13(7)
O(1)-Sm(1)-O(6)	73.92(7)	O(1)-Sm(1)-O(3)	80.05(7)
O(2)-Co(1)-O(9)	108.85(7)	O(1)-Sm(1)-O(8)	142.86(6)
O(4)-Co(1)-O(9)	95.70(7)	O(3)-Sm(1)-O(8)	117.40(7)
O(3)-Sm(1)-O(5)#1	91.65(7)	O(1)-Sm(1)-O(7)#1	75.65(7)
O(8)-Sm(1)-O(5)#1	69.31(6)	O(3)-Sm(1)-O(7)#1	82.65(7)
O(10)-Co(1)-O(9)	57.99(7)	O(8)-Sm(1)-O(7)#1	135.67(6)

O(1)-Sm(1)-O(5)#1	146.53(7)	O(7)#1-Sm(1)-O(9)	147.11(6)
		**8**	
Co(1)-O(9)	2.0096(19)	Co(1)-O(8)	2.0401(18)
Co(1)-N(1)	2.073(2)	Co(1)-O(1)	2.1374(19)
Co(1)-N(2)	2.144(2)	Gd(1)-O(6)#1	2.3580(19)
Gd(1)-O(3)	2.304(2)	Gd(1)-O(2)	2.3752(19)
Gd(1)-O(7)	2.3094(19)	O(4)-Gd(1)#1	2.312(2)
Gd(1)-O(4)#1	2.312(2)	O(6)-Gd(1)#1	2.3580(19)
Gd(1)-O(5)	2.3191(18)	Gd(1)-O(10)	2.3288(18)
O(9)-Co(1)-O(8)	95.90(8)	O(8)-Co(1)-N(2)	169.58(8)
O(9)-Co(1)-O(1)	155.84(7)	O(3)-Gd(1)-O(7)	154.34(8)
O(8)-Co(1)-O(1)	94.35(7)	O(3)-Gd(1)-O(4)#1	122.83(8)
O(3)-Gd(1)-O(5)	79.97(7)	O(7)-Gd(1)-O(4)#1	77.24(8)
O(7)-Gd(1)-O(5)	90.95(7)	O(4)#1-Gd(1)-O(6)#1	73.57(7)
O(4)#1-Gd(1)-O(5)	76.45(8)	O(5)-Gd(1)-O(6)#1	124.28(7)
O(3)-Gd(1)-O(10)	78.79(8)	O(10)-Gd(1)-O(6)#1	138.83(6)
O(7)-Gd(1)-O(10)	76.46(7)	O(3)-Gd(1)-O(2)	101.76(7)
O(4)#1-Gd(1)-O(10)	147.06(7)	O(7)-Gd(1)-O(2)	78.81(7)
O(5)-Gd(1)-O(10)	84.49(7)	O(4)#1-Gd(1)-O(2)	117.46(7)
O(3)-Gd(1)-O(6)#1	78.54(8)	O(5)-Gd(1)-O(2)	159.69(6)
O(7)-Gd(1)-O(6)#1	125.47(8)	O(10)-Gd(1)-O(2)	76.12(7)
		**9**	
Co(1)-O(1)	2.006(2)	Co(1)-N(2)	2.144(2)
Co(1)-O(4)	2.0423(19)	O(2)-Tb(1)	2.3114(18)
Co(1)-N(1)	2.069(2)	O(3)-Tb(1)	2.295(2)
Co(1)-O(8)	2.133(2)	O(5)-Tb(1)	2.300(2)
O(6)-Tb(1)#1	2.293(2)	O(9)-Tb(1)#1	2.3040(19)
O(7)-Tb(1)	2.362(2)	O(10)-Tb(1)	2.3437(19)
O(1)-Co(1)-O(8)	155.41(8)	O(6)#1-Tb(1)-O(5)	122.32(8)
O(4)-Co(1)-O(8)	94.33(8)	O(3)-Tb(1)-O(5)	77.38(8)
O(6)#1-Tb(1)-O(9)#1	79.95(8)	O(5)-Tb(1)-O(2)	147.33(7)
O(3)-Tb(1)-O(9)#1	90.66(8)	O(9)#1-Tb(1)-O(2)	84.52(7)
O(5)-Tb(1)-O(9)#1	76.45(8)	O(6)#1-Tb(1)-O(10)	78.04(8)
O(6)#1-Tb(1)-O(2)	79.02(8)	O(3)-Tb(1)-O(10)	125.99(8)
O(3)-Tb(1)-O(2)	76.53(8)	O(5)-Tb(1)-O(10)	73.30(7)
O(9)#1-Tb(1)-O(10)	123.86(7)	O(5)-Tb(1)-O(7)	117.22(8)
O(2)-Tb(1)-O(10)	138.91(7)	O(9)#1-Tb(1)-O(7)	159.91(7)
		**10**	
Co(1)-O(8)	1.9936(18)	Co(1)-O(3)	2.383(2)
Co(1)-O(2)	2.0389(19)	Dy(1)-O(7)	2.2386(18)
Co(1)-N(2)	2.053(2)	Dy(1)-O(9)#1	2.251(2)
Co(1)-O(4)	2.1004(18)	Dy(1)-O(1)	2.273(2)
Co(1)-N(1)	2.136(2)	Dy(1)-O(10)	2.275(2)
Dy(1)-O(5)#1	2.2909(19)	Dy(1)-O(3)	2.343(2)

Dy(1)-O(6)	2.2958(19)	O(5)-Dy(1)#1	2.2909(19)
O(9)-Dy(1)#1	2.251(2)	O(8)-Co(1)-O(2)	95.34(8)
O(10)-Dy(1)-O(6)	80.20(7)	O(9)#1-Dy(1)-O(3)	116.40(7)
O(5)#1-Dy(1)-O(6)	124.91(7)	O(1)-Dy(1)-O(3)	79.29(7)
O(8)-Co(1)-O(4)	155.06(8)	O(10)-Dy(1)-O(3)	102.13(7)
O(2)-Co(1)-O(4)	95.24(8)	O(5)#1-Dy(1)-O(3)	74.42(7)
O(9)#1-Dy(1)-O(6)	76.67(7)	O(6)-Dy(1)-O(3)	160.19(7)
O(8)-Co(1)-O(3)	98.23(8)	O(4)-Co(1)-O(3)	58.17(7)
O(2)-Co(1)-O(3)	96.14(8)	O(7)-Dy(1)-O(6)	84.08(7)
O(7)-Dy(1)-O(9)#1	147.01(7)	O(1)-Dy(1)-O(10)	155.24(8)
O(7)-Dy(1)-O(1)	77.81(8)	O(7)-Dy(1)-O(5)#1	137.79(7)
O(9)#1-Dy(1)-O(1)	75.85(8)	O(9)#1-Dy(1)-O(5)#1	74.72(7)
O(7)-Dy(1)-O(10)	78.44(8)	O(1)-Dy(1)-O(5)#1	125.54(8)
O(9)#1-Dy(1)-O(10)	123.19(9)	O(10)-Dy(1)-O(5)#1	77.73(8)
**11**			
Co(1)-O(5)	2.006(3)	Ho(1)-O(2)#1	2.262(3)
Co(1)-O(3)	2.040(3)	Ho(1)-O(4)	2.273(3)
Co(1)-N(2)	2.080(3)	Ho(1)-O(10)#1	2.281(3)
Co(1)-O(7)	2.134(3)	Ho(1)-O(1)	2.284(3)
Co(1)-N(1)	2.141(3)	Ho(1)-O(6)	2.291(3)
Ho(1)-O(9)	2.320(3)	O(5)-Co(1)-O(3)	95.65(12)
O(10)-Ho(1)#1	2.281(3)	O(5)-Co(1)-O(7)	154.65(11)
O(2)#1-Ho(1)-O(4)	154.58(12)	O(4)-Ho(1)-O(1)	76.66(10)
O(2)#1-Ho(1)-O(10)#1	80.34(11)	O(10)#1-Ho(1)-O(1)	76.81(11)
O(4)-Ho(1)-O(10)#1	90.11(11)	O(2)#1-Ho(1)-O(6)	78.54(11)
O(2)#1-Ho(1)-O(1)	123.01(11)	O(4)-Ho(1)-O(6)	77.05(11)
O(10)#1-Ho(1)-O(6)	83.88(10)	O(4)-Ho(1)-O(8)	79.16(10)
O(1)-Ho(1)-O(6)	147.14(11)	O(10)#1-Ho(1)-O(8)	159.33(10)
O(10)#1-Ho(1)-O(9)	124.75(10)	O(9)-Ho(1)-O(8)	75.46(9)
O(1)-Ho(1)-O(9)	73.49(11)	O(2)#1-Ho(1)-O(8)	101.93(11)
**12**			
Co(1)-O(9)	1.999(2)	O(2)-Er(1)#1	2.261(2)
Co(1)-O(7)	2.041(2)	O(4)-Er(1)	2.324(2)
Co(1)-N(2)	2.074(3)	O(5)-Er(1)	2.262(2)
Co(1)-O(3)	2.122(2)	O(6)-Er(1)#1	2.250(2)
Co(1)-N(1)	2.141(3)	O(8)-Er(1)	2.261(2)
O(1)-Er(1)	2.311(2)	O(10)-Er(1)	2.277(2)
Er(1)-O(6)#1	2.250(2)	O(8)-Er(1)-O(2)#1	90.14(9)
Er(1)-O(2)#1	2.261(2)	O(9)-Co(1)-O(3)	154.65(9)
O(9)-Co(1)-O(7)	95.90(9)	O(7)-Co(1)-O(3)	94.22(9)
O(8)-Er(1)-O(5)	76.74(9)	O(2)#1-Er(1)-O(10)	83.71(9)
O(2)#1-Er(1)-O(5)	77.01(9)	O(5)-Er(1)-O(10)	147.42(8)
O(6)#1-Er(1)-O(10)	78.48(9)	O(6)#1-Er(1)-O(1)	77.90(9)
O(8)-Er(1)-O(10)	77.31(9)	O(8)-Er(1)-O(1)	125.89(9)

O(2)#1-Er(1)-O(1)	124.52(9)	O(2)#1-Er(1)-O(4)	159.52(8)
O(5)-Er(1)-O(1)	73.48(8)	O(5)-Er(1)-O(4)	116.79(8)
O(10)-Er(1)-O(1)	138.69(8)	O(10)-Er(1)-O(4)	76.90(8)
		**13**	
Co(1)-O(1)	2.001(3)	O(2)-Yb(1)	2.264(3)
Co(1)-O(3)	2.037(3)	O(4)-Yb(1)	2.238(3)
Co(1)-N(1)	2.084(4)	O(6)-Yb(1)	2.297(3)
Co(1)-O(5)	2.101(3)	O(7)-Yb(1)	2.291(3)
Co(1)-N(2)	2.150(4)	O(8)-Yb(1)#1	2.251(3)
O(9)-Yb(1)	2.247(3)	O(8)#1-Yb(1)-O(7)	124.80(12)
O(10)-Yb(1)#1	2.233(3)	O(2)-Yb(1)-O(6)	77.45(12)
Yb(1)-O(10)#1	2.233(3)	O(1)-Co(1)-O(5)	152.78(13)
Yb(1)-O(8)#1	2.251(3)	O(3)-Co(1)-O(5)	95.44(13)
O(1)-Co(1)-O(3)	95.27(13)	O(9)-Yb(1)-O(7)	73.40(14)
O(4)-Yb(1)-O(6)	79.78(13)	O(4)-Yb(1)-O(8)#1	90.68(14)
O(9)-Yb(1)-O(6)	118.30(13)	O(9)-Yb(1)-O(8)#1	76.92(13)
O(8)#1-Yb(1)-O(6)	158.90(11)	O(10)#1-Yb(1)-O(2)	78.00(14)
O(7)-Yb(1)-O(6)	75.50(12)	O(4)-Yb(1)-O(2)	77.50(15)
O(4)-Yb(1)-O(7)	124.91(16)	O(9)-Yb(1)-O(2)	146.28(14)
O(8)#1-Yb(1)-O(2)	82.14(12)	O(2)-Yb(1)-O(7)	140.07(13)

Symmetry transformations used to generate equivalent atoms: #1 -x+1,-y+1,-z for **7**; #1 -x,-y,-z+1for **8**; #1 -x,-y,-z+1 for **9**; #1 -x,-y,-z for **10**; #1 -x+1,-y+2,-z+1 for **11**; #1 -x+1,-y+2,-z+1 for **12**; #1 -x+1,-y,-z for **13**;

Table S3**.** Selected bond distances (Å) and angles (°) for compounds **14**–**16**.

		**14**	
Ho(1)-O(2)	2.239(4)	Ho(1)-O(8)#1	2.270(3)
Ho(1)-O(4)	2.246(3)	Ho(1)-O(10)#1	2.341(5)
Ho(1)-O(6)	2.265(3)	Ho(1)-O(11)	2.537(3)
Co(1)-O(3)	2.051(3)	Co(2)-O(11)	2.149(3)
Ho(1)-O(9)#1	2.077(4)	Co(2)-O(11)#1	2.149(3)
Co(1)-O(5)	2.142(4)	Co(2)-O(9)#1	2.167(3)
Co(1)-O(1)	2.010(3)	Co(2)-O(9)	2.167(3)
Co(2)-O(7)	2.010(3)	O(2)-Ho(1)-O(4)	91.21(15)
O(2)-Ho(1)-O(12)	151.6(4)	O(2)-Ho(1)-O(8)#1	78.99(13)
O(4)-Ho(1)-O(12)	97.2(4)	O(4)-Ho(1)-O(8)#1	78.20(13)
O(2)-Ho(1)-O(6)	79.15(13)	O(5)-Co(1)-O(3)	97.13(14)
O(4)-Ho(1)-O(6)	80.40(14)	O(1)-Co(1)-O(3)	145.05(14)
O(12)-Ho(1)-O(6)	75.6(5)	O(2)-Ho(1)-O(10)#1	87.2(2)
O(2)-Ho(1)-O(11)	152.14(11)	O(8)#1-Ho(1)-O(11)	75.57(11)
O(4)-Ho(1)-O(11)	94.52(12)	O(10)#1-Ho(1)-O(11)	98.01(18)
O(12)-Ho(1)-O(11)	54.2(5)	O(12')-Ho(1)-O(11)	50.6(4)
O(6)-Ho(1)-O(11)	128.68(12)	O(5)-Co(1)-O(1)	95.64(15)

O(4)-Ho(1)-O(9)#1	152.35(12)	O(12)-Ho(1)-O(9)#1	88.6(5)
		**15**	
Er(1)-O(4)	2.234(4)	Er(1)-O(10)#1	2.328(4)
Er(1)-O(6)	2.262(3)	Er(1)-O(12)	2.540(3)
Er(1)-O(7)#1	2.269(3)	Er(1)-O(9)#1	2.594(3)
Er(1)-O(11)	2.319(14)	Co(1)-O(5)	2.013(3)
Co(1)-O(3)	2.042(3)	Co(2)-O(8)#1	2.012(3)
Co(1)-O(1)	2.046(3)	Co(2)-O(12)#1	2.160(3)
Co(1)-N(1)	2.085(3)	Co(2)-O(12)	2.160(3)
Co(1)-N(2)	2.143(3)	Co(2)-O(9)#1	2.165(3)
Co(2)-O(8)	2.012(3)	Co(2)-O(9)	2.165(3)
O(4)-Er(1)-O(6)	79.54(13)	O(2)-Er(1)-O(11)	97.1(3)
O(2)-Er(1)-O(6)	80.37(12)	O(6)-Er(1)-O(11)	76.4(3)
O(4)-Er(1)-O(7)#1	78.71(12)	O(7)#1-Er(1)-O(11)	128.6(3)
O(2)-Er(1)-O(7)#1	78.32(12)	O(4)-Er(1)-O(10)#1	87.3(2)
O(6)-Er(1)-O(7)#1	148.83(11)	O(2)-Er(1)-O(10)#1	156.22(14)
O(4)-Er(1)-O(12)	151.81(11)	O(2)-Er(1)-O(9)#1	152.11(10)
O(2)-Er(1)-O(12)	94.40(11)	O(6)-Er(1)-O(9)#1	127.39(10)
O(6)-Er(1)-O(12)	128.61(11)	O(7)#1-Er(1)-O(9)#1	76.95(10)
O(7)#1-Er(1)-O(12)	75.75(10)	O(11)-Er(1)-O(9)#1	88.4(3)
O(3)-Co(1)-O(1)	144.65(13)	O(8)#1-Co(2)-O(9)#1	93.31(11)
O(8)#1-Co(2)-O(12)	91.98(11)	O(12)-Co(2)-O(9)#1	82.02(11)
O(12)#1-Co(2)-O(12)	180.000(1)	O(8)-Co(2)-O(9)	93.31(11)
		**16**	
Yb(1)-O(2)	2.225(3)	Yb(1)-O(7)	2.458(3)
Yb(1)-O(6)	2.228(3)	Yb(1)-O(11)	2.612(3)
Yb(1)-O(9)	2.236(3)	Yb(1)-Co(2)	3.6353(16)
Yb(1)-O(12)	2.293(5)	Co(1)-O(5)	2.005(3)
Yb(1)-O(8)	2.382(4)	Co(1)-O(3)	2.040(3)
Yb(1)-O(12')	2.398(15)	Co(1)-O(1)	2.052(3)
Yb(1)-O(4)	2.198(4)	Co(1)-N(1)	2.076(4)
Co(1)-N(2)	2.139(4)	Co(2)-Yb(1)#1	3.6353(16)
Co(2)-O(10)#1	2.014(3)	O(4)-Yb(1)-O(2)	92.90(13)
Co(2)-O(10)	2.014(3)	O(4)-Yb(1)-O(6)	78.99(12)
Co(2)-O(7)#1	2.141(3)	O(2)-Yb(1)-O(6)	81.53(11)
Co(2)-O(7)	2.141(3)	O(4)-Yb(1)-O(9)	79.31(12)
Co(2)-O(11)	2.150(3)	O(2)-Yb(1)-O(9)	76.73(11)
Co(2)-O(11)#1	2.150(3)	O(6)-Yb(1)-O(9)	148.26(12)
O(4)-Yb(1)-O(12)	95.11(19)	O(9)-Yb(1)-O(8)	124.20(12)
O(2)-Yb(1)-O(12)	154.33(16)	O(12)-Yb(1)-O(8)	75.4(2)
O(6)-Yb(1)-O(12)	76.15(15)	O(7)#1-Co(2)-O(7)	180.000(1)
O(9)-Yb(1)-O(12)	128.74(15)	O(10)#1-Co(2)-O(11)	86.29(12)
O(4)-Yb(1)-O(8)	155.67(12)	O(10)-Co(2)-O(11)	93.71(12)
O(2)-Yb(1)-O(8)	87.47(14)	O(7)#1-Co(2)-O(11)	97.76(11)

O(6)-Yb(1)-O(8)	77.01(12)	O(7)-Co(2)-O(11)	82.24(11)
O(4)-Yb(1)-O(11)	90.50(11)	O(5)-Co(1)-O(3)	96.08(12)
O(2)-Yb(1)-O(11)	152.07(10)	O(5)-Co(1)-O(1)	97.46(12)
O(6)-Yb(1)-O(11)	126.27(10)	O(3)-Co(1)-O(1)	142.64(13)
O(9)-Yb(1)-O(11)	76.69(10)	O(10)#1-Co(2)-O(10)	180.0
O(12)-Yb(1)-O(11)	52.27(14)	O(10)#1-Co(2)-O(7)	88.26(11)
O(8)-Yb(1)-O(11)	100.48(12)	O(10)#1-Co(2)-O(11)#1	93.71(12)
O(12')-Yb(1)-O(11)	51.6(4)	O(10)-Co(2)-O(11)#1	86.29(12)
O(7)-Yb(1)-O(11)	67.57(9)	O(10)#1-Co(2)-O(7)#1	91.74(11)

Symmetry transformations used to generate equivalent atoms: #1 -x+2,-y+1,-z+1 for **14**; #1 -x+1,-y+2,-z+1 for **15**; #1 -x+1,-y,-z+1 for **16**.

Table S4. The Crystal Data for **17** and **18**

Compound	**17**	**18**
Empricial formula	$C_{69}H_{44}Cl_7Co_2N_4O_{14}Y$	$C_{90}H_{46}Cl_{20}Co_2N_4O_{20}Y_2$
Fw	1608.00	2507.99
temp (K)	293(2)	293(2)
crystal syst.	Triclinic	Triclinic
space group	$P\bar{1}$	$P\bar{1}$
a(Å)	11.765(2)	13.508(3)
b(Å)	13.594(3)	13.821(3)
c(Å)	21.497(4)	13.887(3)
α (˚)	80.08(3)	99.83(3)
β (˚)	89.69(3)	95.70(3)
γ (˚)	89.79(3)	107.57(3)
V (Å3)	3386.6(11)	2403.8(9)
Z	2	1

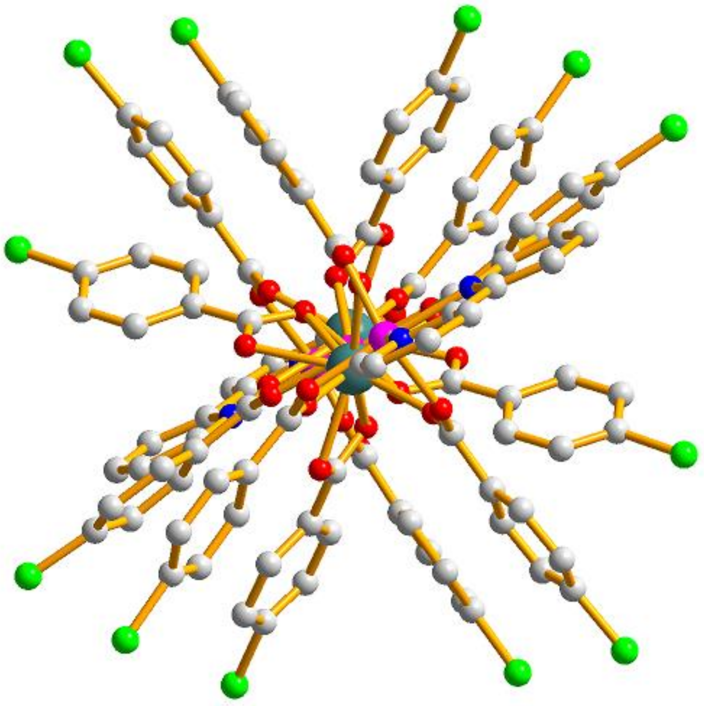

Figure S1. The paddlewheel arrangement of the ligands viewed along the Co1-Ho1-Co2-Ho1a-Co1a axis in **14**.

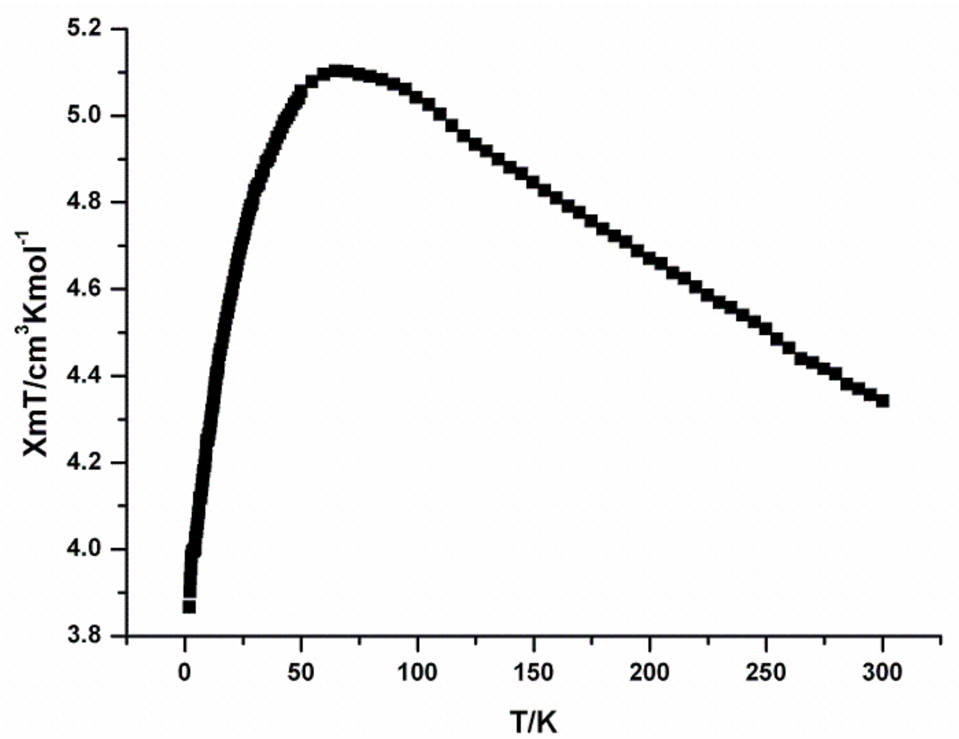

Figure S2. Temperature dependence of the χ_mT curves at 1000Oe for the compound **17**.

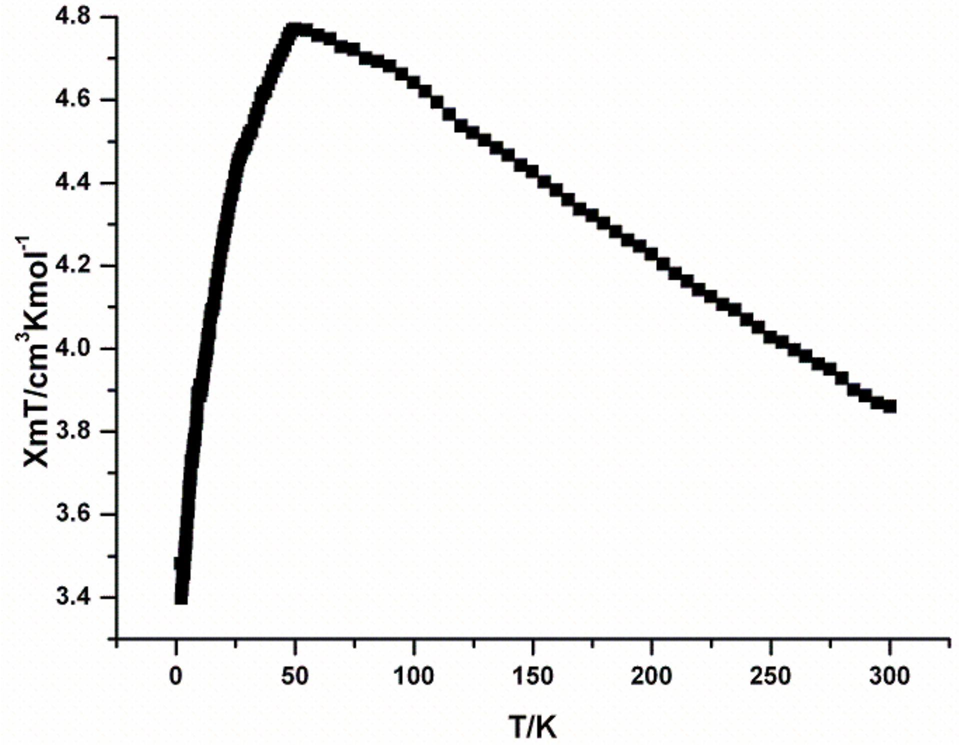

Figure S3. Temperature dependence of the χ_mT curves at 1000Oe for the compound **18**

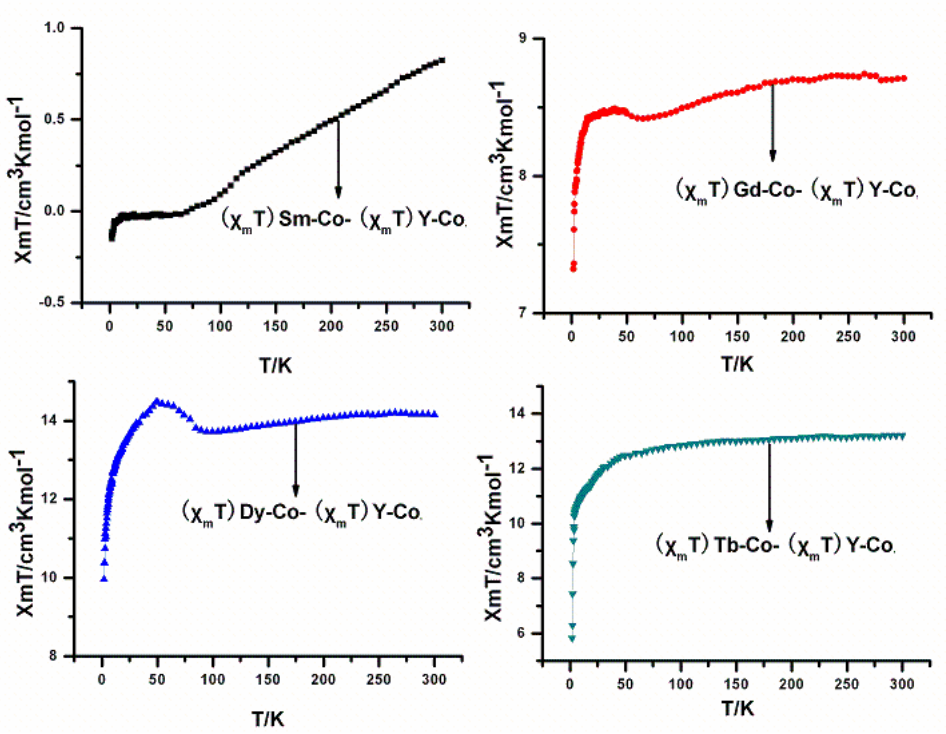

Figure S4. The Result of the subtraction of the Co^{2+} paramagnetic contribution of complexes **3-6**

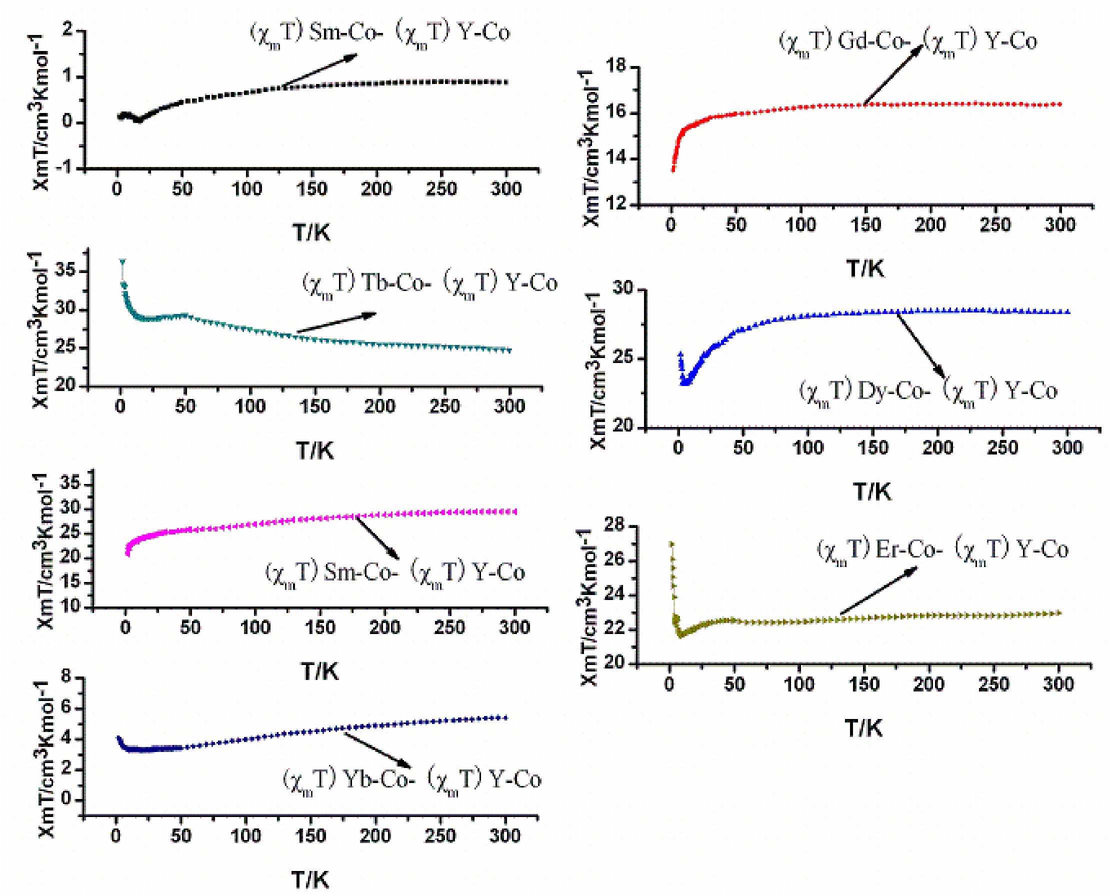

Figure S5. The Result of the subtraction of the Co^{2+} paramagnetic contribution of

complexes **7-13**

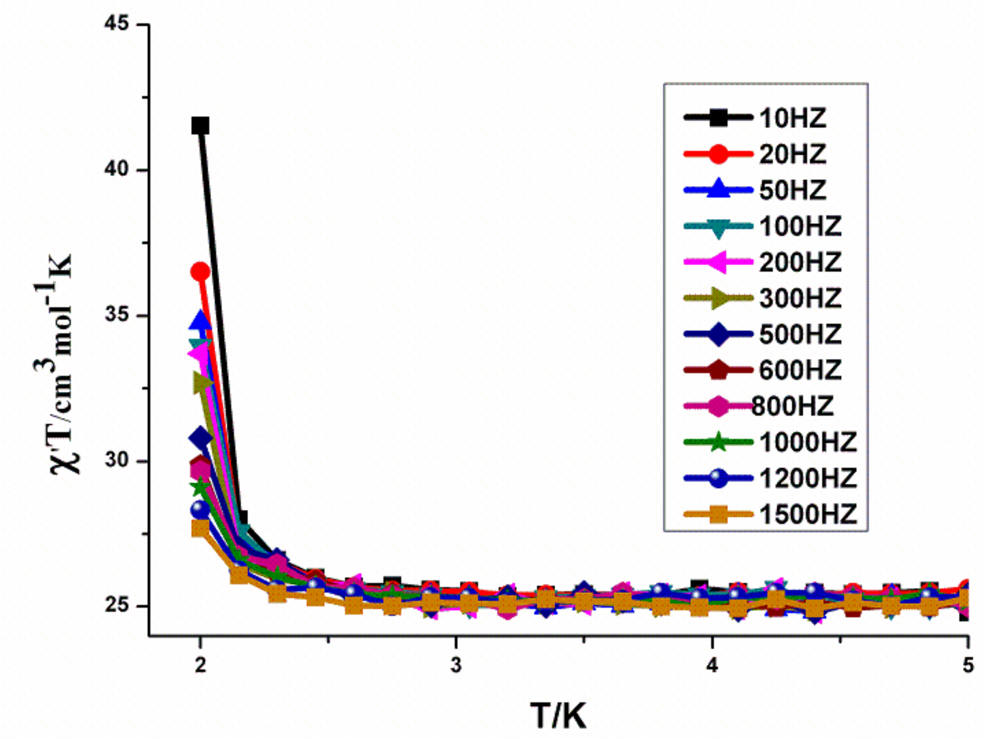

Figure S6. The temperature dependence of in-phase component of the ac susceptibility at different frequencies of compound **14**

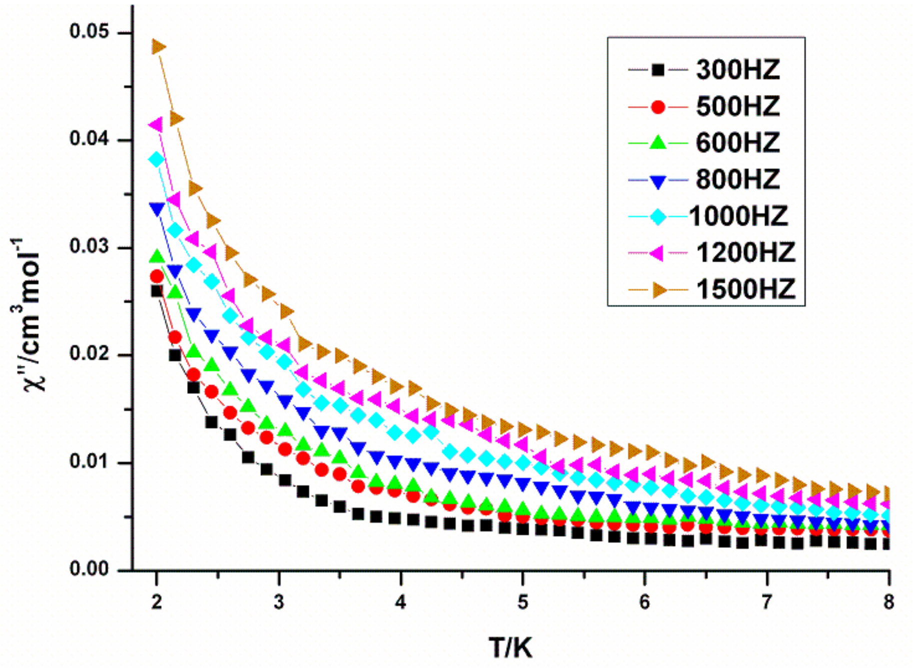

Figure S7. The temperature dependence of out-of-phase component of the ac susceptibility at different frequencies of compound **14**
